# K-bZIP Mediated SUMO-2/3 Specific Modification on the KSHV Genome Negatively Regulates Lytic Gene Expression and Viral Reactivation

**DOI:** 10.1371/journal.ppat.1005051

**Published:** 2015-07-21

**Authors:** Wan-Shan Yang, Hung-Wei Hsu, Mel Campbell, Chia-Yang Cheng, Pei-Ching Chang

**Affiliations:** 1 Institute of Microbiology and Immunology, National Yang-Ming University, Taipei, Taiwan, Republic of China; 2 UC Davis Cancer Center, University of California, Davis, Davis, California, United States of America; 3 Center for Infectious Disease and Cancer Research, Kaohsiung Medical University, Kaohsiung, Taiwan, Republic of China; University of Southern California, UNITED STATES

## Abstract

SUMOylation is associated with epigenetic regulation of chromatin structure and transcription. Epigenetic modifications of herpesviral genomes accompany the transcriptional switch of latent and lytic genes during the virus life cycle. Here, we report a genome-wide comparison of SUMO paralog modification on the KSHV genome. Using chromatin immunoprecipitation in conjunction with high-throughput sequencing, our study revealed highly distinct landscape changes of SUMO paralog genomic modifications associated with KSHV reactivation. A rapid and widespread deposition of SUMO-2/3, compared with SUMO-1, modification across the KSHV genome upon reactivation was observed. Interestingly, SUMO-2/3 enrichment was inversely correlated with H3K9me3 mark after reactivation, indicating that SUMO-2/3 may be responsible for regulating the expression of viral genes located in low heterochromatin regions during viral reactivation. RNA-sequencing analysis showed that the SUMO-2/3 enrichment pattern positively correlated with KSHV gene expression profiles. Activation of KSHV lytic genes located in regions with high SUMO-2/3 enrichment was enhanced by SUMO-2/3 knockdown. These findings suggest that SUMO-2/3 viral chromatin modification contributes to the diminution of viral gene expression during reactivation. Our previous study identified a SUMO-2/3-specific viral E3 ligase, K-bZIP, suggesting a potential role of this enzyme in regulating SUMO-2/3 enrichment and viral gene repression. Consistent with this prediction, higher K-bZIP binding on SUMO-2/3 enrichment region during reactivation was observed. Moreover, a K-bZIP SUMO E3 ligase dead mutant, K-bZIP-L75A, in the viral context, showed no SUMO-2/3 enrichment on viral chromatin and higher expression of viral genes located in SUMO-2/3 enriched regions during reactivation. Importantly, virus production significantly increased in both SUMO-2/3 knockdown and KSHV K-bZIP-L75A mutant cells. These results indicate that SUMO-2/3 modification of viral chromatin may function to counteract KSHV reactivation. As induction of herpesvirus reactivation may activate cellular antiviral regimes, our results suggest that development of viral SUMO E3 ligase specific inhibitors may be an avenue for anti-virus therapy.

## Author Summary

Establishment of KSHV persistent infection requires a dynamic balance between latency, a phase where most viral genes are silenced, and lytic cycle, a phase when nearly all viral genes are expressed. Disruption of this balance may augment virus clearance. During the latent-to-lytic switch, KSHV genomes are subjected to profound epigenetic changes. SUMOylation promotes targeting of proteins to different DNA sites, thereby helping to create specific epigenetic patterns that switch genes between active and inactive stages. It comes as no surprise that SUMOylation may be involved in chromatin remodeling of the KSHV genome during the latent-to-lytic switch and SUMOylation inhibition may disrupt the balance between KSHV latent and lytic cycle. In this study, we identified a profound SUMO-2/3 enrichment in KSHV genome euchromatin regions upon reactivation. SUMO-2/3 modification is responsible for diminishing viral gene expression after reactivation. KSHV SUMO-2/3-specific E3 ligase K-bZIP mediates the SUMO-2/3 enrichment during reactivation. Loss of E3 ligase activity of K-bZIP in the viral context increases viral lytic gene expression and virus production. Our findings demonstrate, for the first time, a SUMO-2/3-specific modification affecting transcription which regulates viral lytic gene expression, and uncovers a novel therapeutic strategy to disrupt persistent infection.

## Introduction

Dynamic chromatin structure regulation by post-translational protein modifications modulates the accessibility of DNA and consequently the transcription of genes. Small ubiquitin-like modifier (SUMO) modification in epigenetic regulation of chromatin states has been intensively studied. SUMO modification of specific transcription factors or chromatin remodeling proteins, in most cases, is associated with repressive complex formation and a silencing role in transcription regulation [1,2]. Moreover, SUMOylation promotes *de novo* targeting of chromatin proteins to heterochromatin [3]. However, increasing evidence suggests that SUMO modification may also be associated with positive regulation of transcription [4]. These data highlight the complexity of chromatin-associated SUMO in gene expression modulation. To uncover the global epigenetic role of SUMO in transcription regulation, one study performed in yeast showed that SUMO associates with promoters of constitutively active and inducible genes. SUMO recruitment to inducible promoters during activation is required to shut-off inducible genes after elimination of the activating signal [5]. Unlike yeast, that contains only a single SUMO protein, human cells have three protein-conjugating isoforms. These isoforms include SUMO-1, which is conjugated to proteins as a monomer, and highly related SUMO-2 and SUMO-3 (SUMO-2/3), which are known to form high molecular weight polymers on proteins [6,7]. Though earlier studies have pinpointed some important differences between SUMO-1 and SUMO-2/3 [8,9], the functional specificity of SUMO isoforms in global epigenetic regulation of gene expression is just beginning to be uncovered. Several recent reports, including ours, using Chromatin Immunoprecipitation-Sequencing (ChIP-seq) in combination with transcriptome analysis of RNA-sequencing (RNA-seq) have comprehensively characterized the SUMO-1 and SUMO-2/3 genomic landscape and their global role in transcription regulation in human cells [10–12]. Neyret-Kahn *et al* showed that both SUMO-1 and SUMO-2/3 are strongly associated at promoters of actively transcribed genes and SUMOylation is responsible for restraining their expression during cell proliferation [12]. In contrast, Liu *et al* showed that SUMO-1 is associated with promoters of active genes and directly activates their transcription during interphase of the cell cycle [11]. The association of SUMO with the active histone mark H3K4me3 was identified in both studies. However, similar epigenetic alterations between SUMO paralogs were observed under physiological stimuli tested [12]. Interestingly, our recent report showed that SUMO-2/3, when compared with SUMO-1 modifications around cellular promoter regions was significantly increased in B cells during Kaposi’s sarcoma associated herpesvirus (KSHV) reactivation. This enrichment prevents the activation of host genes during viral reactivation [10]. These findings indicate the existence of differential roles of SUMO paralogs in regulating chromatin and transcription during a stress response, such as virus infection.

KSHV, also known as human herpesvirus type 8 (HHV-8), is a γ-herpesvirus associated with Kaposi’s sarcoma (KS), a tumor of endothelial origin, and primary effusion lymphomas (PEL), a B-cell lymphoma [13]. Similar to all herpesviruses, the KSHV lifecycle has distinct latent and lytic phases. KSHV can maintain a tightly latent infection in the majority of infected tumor cells. However, a small population of infected cells continues to undergo spontaneous lytic replication [14]. Establishing latency enables KSHV to evade host immune surveillance, establish persistent life-long infections and induce tumorigenesis [15]. Lytic reactivation is not only required for the proper spread of KSHV infection, but is also a prerequisite for the maintenance of a population of latently infected cells and KSHV pathogenesis [16]. After infection, KSHV genomic DNA in the host cells forms a chromatin-like structure. The latent–to-lytic switch involves global remodeling of viral chromatin from the heterochromatin to the euchromatin state. Recently, several studies including ours have begun to document the histone modification profiles on KSHV viral chromatin during viral infection or reactivation [17–20]. These studies indicate that activating and repressive histone marks are differentially located on KSHV latent and lytic genomes and these marks are involved in transcriptional regulation of viral genes. The bivalent states of chromatin marks on the KSHV-replication and transcription activator (K-Rta) promoter maintains a poised state for K-Rta rapid expression in response to reactivation stimuli. Latent genes possess only activating histone marks. Most early genes have either activating or repressive histone marks. The difference between chromatin modifications may contribute to the temporal expression of KSHV genes during reactivation from latency. However, one area that is complex and remains largely unknown is the function of additional post-translational modifications, such as SUMOylation, in regulating the viral epigenome.

Analogous to ubiquitylation, SUMOylation is a multistep enzyme cascade including SUMO E1 activating enzyme (SAE1/SAE2), SUMO E2 conjugating enzyme (Ubc9), and SUMO E3 ligase (i.e., PIAS family, RanBP2, and Pc2). However, unlike ubiquitylation, an E3 ligase is not essential for SUMO conjugation, but instead provides specificity for SUMO modification. The SUMO interaction motifs (SIMs) in SUMO E3 ligases are responsible for its SUMO paralog specificity [21,22] and structure analysis shows potentially different specificity of SIMs toward SUMO paralogs [23]. This underlying complexity was increased by the identification of the downstream consequences of non-covalent interaction with effectors via SIMs, providing an additional interaction platform for selectively recruiting SUMO-1 or SUMO-2/3 specific SIM-containing proteins. As mentioned earlier, SUMO modification of chromatin proteins may formulate the fine-tuning of chromatin structure and transcription regulation. Like other DNA viruses, KSHV has evolved different mechanisms to directly or indirectly manipulate the SUMO machinery to advance their own growth (reviewed in [24–26]). Most interestingly, we recently identified KSHV lytic protein K-bZIP as a SUMO E3 ligase with specificity toward SUMO-2/3 [27]. This unique specificity suggests the potential preferential usage of SUMO-2/3 by KSHV to globally modulate its epigenome and gene expression during lytic reactivation. Hence, KSHV represents an ideal model system to study the functional specificity of SUMO-2/3 in regulating global epigenetic changes and transcription. Moreover, this specificity makes KSHV an attractive model system to help distinguish SUMO paralog-specific effects in epigenetic regulation of transcription.

In this study, we demonstrate SUMO-2/3 specific chromatin modification enrichment on the KSHV genome during lytic reactivation. RNA-seq results show a positive correlation between viral lytic gene transcription activation and SUMO-2/3 enrichment on the viral genome upon reactivation. SUMO-2/3 knockdown results in increased transcription of viral lytic genes located in high SUMO-2/3 enrichment regions of the KSHV genome. Interestingly, the overlaid SUMO-2/3 binding pattern and different epigenetic marks showed a positive correlation between SUMO-2/3 with the active histone mark H3K4me3 and a negative correlation between SUMO-2/3 with the repressive histone mark H3K27me3 in the latent viral genome. In addition, a negative correlation between SUMO-2/3 enrichment and H3K9me3 marks in viral lytic genomes during the early phase of KSHV reactivation was identified. These results suggest that SUMO-2/3 modification plays an essential role in fine-tuning genomic regions with active chromatin structure but not repressive heterochromatin regions. Since KSHV encodes a SUMO-2/3 specific E3 ligase, K-bZIP, we analyzed K-bZIP binding on the KSHV genome by a ChIP assay. A significant increase of K-bZIP binding in SUMO-2/3 enrichment region after KSHV reactivation was found. In addition, we used the BAC16 template to generate a new recombinant BACmid, BAC16 K-bZIP-L75A, which contains a SIM domain mutant of K-bZIP that has lost its SUMO E3 ligase activity. Cell lines stably transfected with BAC16 established latency and could produce infectious virus upon reactivation. The K-bZIP SUMO E3 ligase dead mutant showed increased expression level of viral lytic transcripts located in high SUMO-2/3 enriched regions and produced significantly more infectious viruses. These data strongly suggest SUMO-2/3 specific epigenetic regulation of viral gene expression during reactivation.

## Materials and Methods

### Cell culture

The doxycycline (Dox)-inducible TREx-BCBL-1, 3x Flag- and 3x His-tagged K-Rta BCBL-1 (TREx-F3H3-K-Rta BCBL-1) and Myc-His-tagged K-Rta BCBL-1 (TREx-MH-K-Rta BCBL-1) cell lines were maintained in RPMI 1640 containing 15% FBS, 50 μg/ml blasticidin and 100 μg/ml Zeocin or 100 μg/ml hygromycin (Invitrogen, Carlsbad, CA). TREx-BCBL-1, TREx-F3H3-K-Rta BCBL-1 and TREx-MH-K-Rta BCBL-1 cells were induced with 0.2 μg/ml Dox. The SUMO-2/3 inducible knockdown cell line was generated in previous study [10]. Briefly, the shSUMO-2 and shSUMO-3 in pLenti4-H1/TO-shRNA plasmid were introduced into TREx-F3H3-K-Rta BCBL-1 cells by transduction and the stable TREx-F3H3-K-Rta-shSUMO-2/3 BCBL-1 cell line was maintained as described for TREx-F3H3-K-Rta BCBL-1 cells and supplemented with 300 μg/ml Zeocin. Induction of SUMO knockdown and K-Rta expression was confirmed by immunoblotting analysis. The SUMO-1 and SUMO-2 overexpression cell lines were generated by transfection using plasmids expressing Flag-SUMO-1 or Flag-SUMO-2 into TREx-MH-K-Rta BCBL-1 cells. Cells were selected for 21 days by 200 μg/ml G418 (AMRESCO) and purified by Ficoll. Expression of Flag-tagged SUMO-1 and SUMO-2 were tested by immunoblotting using anti-Flag antibody. iSLK-Puro cells were maintain in DMEM containing 10% FBS, 250 μg/ml G418 and 1 μg/ml puromycin (Invitrogen). 293T cells were maintained in DMEM containing 10% FBS.

### Chromatin Immunoprecipitation-Sequencing (ChIP-Seq) and real-time quantitative PCR (qPCR)

ChIP was performed using the protocol from Dr. Farnham’s laboratory (http://genomics.ucdavis.edu/farnham). Briefly, chromatin DNA from control and Dox-treated TREx-F3H3-K-Rta BCBL-1 cells were harvested. Chromatin DNA from 1 x 10^7^ cells was used per antibody for each ChIP assay. ChIP grade anti-SUMO-1 (Abcam, ab32058) and anti-SUMO-2/3 (Abcam, ab3742) specific rabbit polyclonal antibodies, as well as rabbit non-immune serum IgG (Alpha Diagnostic International), were used for the ChIP assays. 50 ng of ChIPed DNA suspended in 30 μl of ddH_2_O was used for ChIP-seq library preparation following the protocol from Illumina. Size-selected (400 bp) DNA fragment libraries were used for paired-end high throughput sequencing on Illumina Genome Analyzer_*II*_. The ChIP-Seq data was aligned with the KSHV genome build by Avadis NGS (Strand Scientific Intelligence, San Francisco, CA). Approximately 1~3 x 10^6^ reads were mapped for each sample after filtering and quality control (QC). In this study, we used the target region QC detection method of Avadis NGS to delineate the SUMO-1 and SUMO-2/3 binding patterns. ChIP DNA was verified for successful IP by SYBR Green-Based real-time qPCR using CFX connect real-time PCR detection system (Bio-Rad, Richmond, CA). Specific primer sets were designed around the potential binding sites. Primer sequences were listed in S1 Table.

### RNA-seq and reverse transcription-qPCR (RT-qPCR) analysis

Total RNA was prepared from TREx-F3H3-K-Rta BCBL-1 cells harvested at 0, 12 and 24 hours after Dox treatment using TRIzol (Invitrogen, Carlsbad, CA) according to the manufacturer’s instructions. RNA-seq was carried out at the Sequencing Core of National Research Program for Genomic Medicine at the National Yang-Ming University using an Illumina Genome Analyzer_*II*_. Sequencing reads were processed as described previously [10]. In this study, the sequence reads that did not align with hg19 were mapped to KSHV. The transcript abundances were estimated in reads per kilobase of transcript per million mapped reads (RPKM) by Avadis NGS. Differential gene expression was analyzed by comparing RPKMs from each sample and verified using real-time RT-qPCR. 2 μg of total RNA was reverse-transcribed using Oligo-d(T)_18_ and SuperScript III first-strand synthesis system (Invitrogen). qPCR was carried out according to the manufacturer's protocol (iQ SYBR Green Supermix, Bio-Rad). Primer sequences were listed in S2 Table.

### Generation of K-bZIP-L75A mutant and wild-type revertant BAC16

To mutate K-bZIP Leu 75 to Ala within BAC16 using recombineering, we first generated a targeting vector containing a KpnI/HindIII fragment of the KSHV genome that included partial K-Rta coding region and the complete K-bZIP coding region. The primer 5’-GGTCTGTGAAACGGTCATTGACGCTACAGCGCCTTCCCAAA-3’ containing the L75A mutation flanked by 14~15 bp homology were used to target mutagenesis of K-bZIP at Leu 75. After confirmation of mutation, the FRT-flanked kanamycin cassette was inserted into the SalI site in between the K-Rta and K-bZIP coding region. A linear fragment for homologous recombination was generated by digestion targeting vector with KpnI and HindIII. The DNA fragment was gel purified by RECOCHIP (Takara) and electroporated into induced (recombination +) SW105 harboring BAC16. Kanamycin resistant colonies were selected and the insertion of the targeting cassette was confirmed by PCR. For kanamycin cassette removal, positive colonies were inoculated in LB containing arabinose and incubated overnight at 32°C. Kanamycin-sensitive clones were further screened by PCR. After successful removal of the kanamycin cassette in K-bZIP-L75A mutants, clones were verified by sequencing. To make a wild-type (WT) revertant, K-bZIP-L75A in BAC16 was replaced by WT K-bZIP using the recombineering protocol as described above.

### Southern blot analysis

KpnI and HindIII cleaved BAC16 DNA was separated on 1% agarose gel and visualized by ethidium bromide staining. The DNA in the gel was transferred to NC membrane using downward alkaline transfer. A K-bZIP probe was radiolabeled with [α-^32^P] dCTP (Perkin Elmer) using Rediprime II random primer labeling kit (GE Healthcare, UK). DNA blots were hybridized overnight at 65°C with rotation. After washing, the blots were imaged using X-ray film (Kodak, Rochester, NY, USA).

### Generation of iSLK-Puro-BAC16 stable cell lines

iSLK-Puro cells were transfected with 2 μg of BAC16 DNA using FuGENE HD (Roche). Forty-eight hours after transfection, the cells were expanded to a 15 cm Petri dish and selected by hygromycin (600 μg/ml). After three weeks of selection, the hygromycin-resistant and GFP positive colonies were picked and pooled to establish the iSLK-Puro-BAC16 K-bZIP-WT, -WT rev and -L75A cell lines. iSLK-Puro-BAC16 cell lines were maintained as described for iSLK-Puro cells and supplemented with 300 μg/ml hygromycin.

### Quantification of KSHV virions by TaqMan qPCR

To assess viral production, supernatants from control and Dox-induced TREx-F3H3-K-Rta BCBL-1 and SUMO-2/3 knockdown TREx-F3H3-K-Rta-shSUMO-2/3 BCBL-1 cells were collected before and after 48 hours treatment. KSHV virion DNA was prepared using QIAamp MinElute Virus Spin kits as described previously [28]. Quantification was performed by real-time qPCR using a TaqMan probe targeting orf73 (LANA) [29].

## Results

### Distinct chromatin binding patterns of SUMO-1 and SUMO-2/3 modifications on the KSHV genome during reactivation

To distinguish the genome-wide distribution of SUMO paralog conjugation on KSHV chromatin and determine their changes during viral reactivation, we conducted chromatin immunoprecipitation (ChIP) assays in combination with high-throughput next generation sequencing (ChIP-seq), a gold-standard method for identifying the genome-wide sites of epigenetic marks. For this, we used the well-characterized KSHV-infected primary effusion lymphoma (PEL) cell line, TREx-F3H3-K-Rta BCBL-1, that expressed a Dox inducible K-Rta protein whose expression switches KSHV from latent to lytic phase [30]. The induction of K-bZIP during KSHV reactivation by Dox induced K-Rta overexpression or by PKC agonist TPA treatment was first compared. A comparable level of K-bZIP expression was identified in cells receiving 0.2 μg/ml Dox or 20 ng/ml TPA treatment (S1 Fig). Therefore, 0.2 μg/ml of Dox was used to treat K-Rta inducible TREx-F3H3-K-Rta BCBL-1 cells for our entire study to prevent induction of excessively high levels of K-bZIP by high dose Dox treatment.

**Fig 1:**
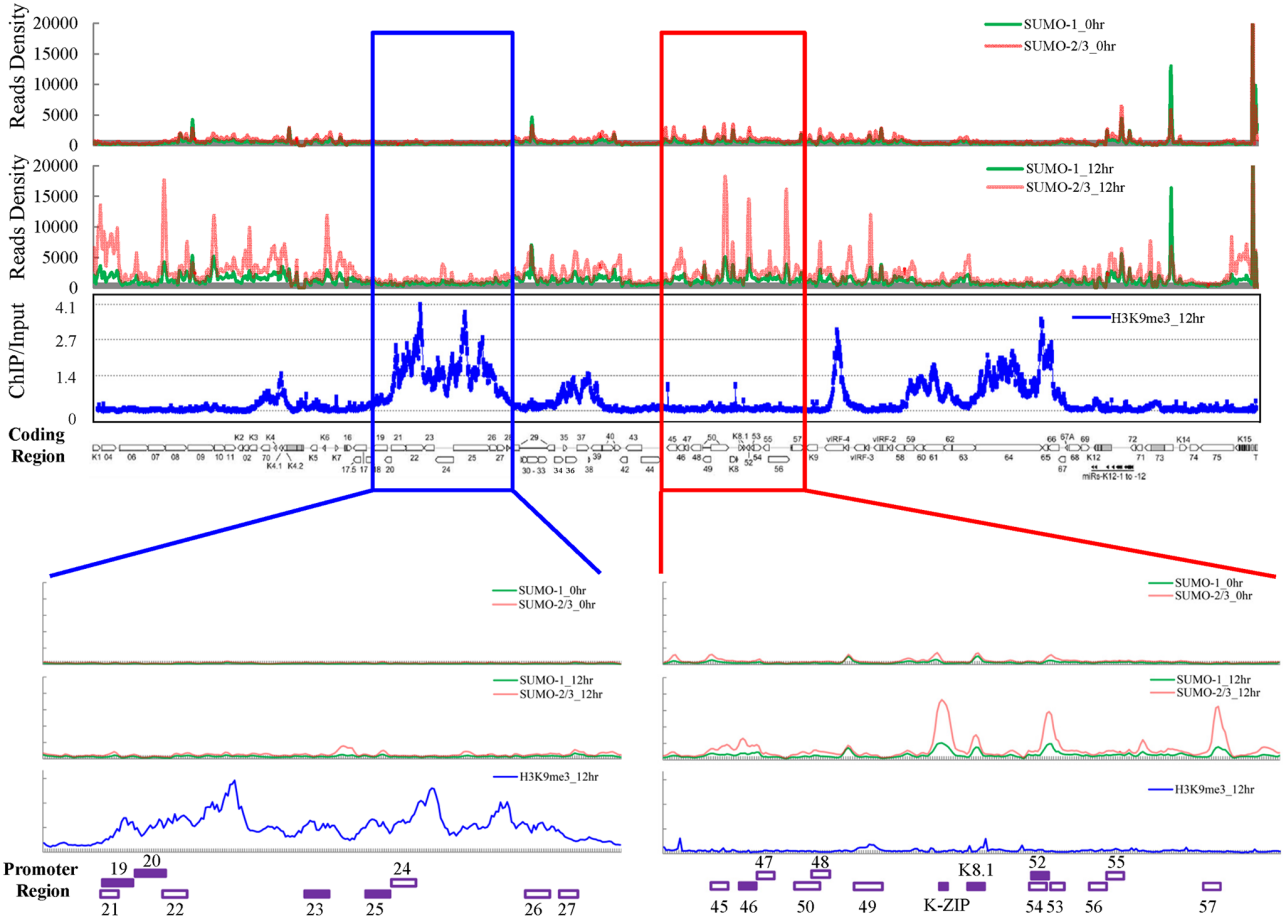
Global mapping of SUMO paralogs on latent and lytic KSHV genomes and of H3K9me3 on the KSHV genome during lytic reactivation. ChIP-seq for SUMO paralogs was performed using chromatin prepared from non-induced (0 hour) and 0.2 μg/ml Dox-treated (12 hours) TREx-F3H3-K-Rta BCBL-1 cells. The ChIP-seq result of the KSHV genome was normalized with total reads from the human genome. ChIP-on-chip for H3K9me3 was performed using chromatin prepared from 0.2 μg/ml Dox-treated cells as described above. The rectangular areas are regions that comprise high SUMO-2/3 (red) or high H3K9me3 (blue) levels after KSHV reactivation (upper panel) and are further magnified on the lower portion of the figure. Promoters of viral genes in these regions are marked in bottom. Solid squares represent genes used for further analysis (lower panel).

After Dox treatment for 12 hours, the global expression of SUMO-1 and SUMO-2/3 and successful induction of K-Rta were first confirmed by immunoblotting (S2A Fig). The ChIP experiments were carried out using chromatin prepared from non-induced (latency phase) and Dox-induced (lytic phase) TREx-F3H3-K-Rta BCBL-1 cells. Next generation sequencing was then performed to measure the chromatin binding of SUMO-1 and SUMO-2/3 from a single run of ChIP assay. As shown in Fig 1, the ChIP-seq result revealed a comparable binding level of SUMO-1 and SUMO-2/3 throughout the KSHV latent genome. Interestingly, SUMO-2/3 enrichment levels were significantly increased on most parts of the KSHV genome at 12 hours post induction (hpi) of viral reactivation when compared to the changes observed in SUMO-1 levels (Fig 1). However, it should be noted that a significant higher enrichment of SUMO-1 than SUMO-2/3 was observed in a few regions of the viral genome, including the promoter of latent gene orf73 (LANA). The potential role of this SUMO-1 specific enrichment in regulation LANA gene expression is interesting and a subject for further inquiry. However, in this study, we focused on studying the global enrichment of SUMO-2/3 in regulating viral gene expression and reactivation.

Surprisingly, when compared with our previous result of H3K9me3 modification on the KSHV latent genome (0 hpi) [17], we noticed that two distinct viral genomic regions, which contain a high level of the repressive heterochromatin mark H3K9me3, displayed no increase of SUMO-2/3 occupancy at 12 hpi. To further investigate whether SUMO-2/3 enrichment at 12 hpi is indeed negatively correlated with H3K9me3, we performed a ChIP-on-chip experiment by hybridizing DNA from H3K9me3-associated chromatin at 12 hpi (Fig 1) with a KSHV genome tiling array we previously designed [17]. We first aligned the two binding profiles at 0 and 12 hpi on the KSHV genome and then analyzed the correlation by Pearson Correlation analysis. Applying this measure, a medium negative correlation (r = -0.3~-0.5) was identified between SUMO-2/3 modification and H3K9me3 at 12 hpi (r = -0.3) but not at 0 hpi (r = -0.1). These data suggest that SUMO-2/3 specific modification appears to have an epigenetic regulatory function separate from H3K9me3.

**Fig 2:**
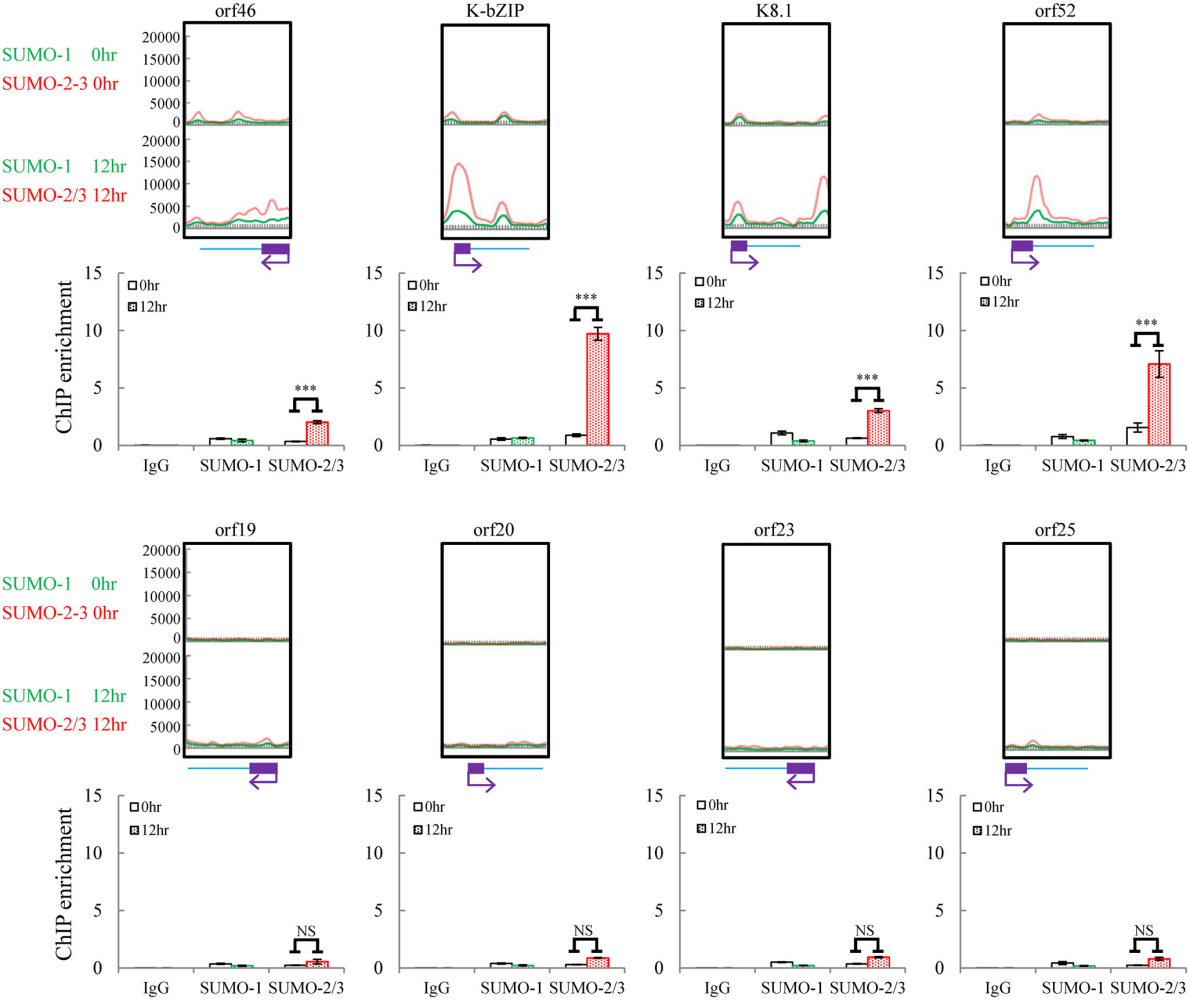
Deposition of SUMO-2/3 modification on selected KSHV promoters representing high SUMO-2/3 enrichment or high H3K9me3 mark regions during reactivation. ChIP was performed as described in Fig 1 using anti-SUMO-1 or anti-SUMO-2/3 antibodies. SUMO-1 and SUMO-2/3 binding to orf46, K-bZIP, K8.1, and orf52 promoters in SUMO-2/3 enrichment region and orf19, orf20, orf23, and orf25 promoters in H3K9me3-rich region were analyzed by real-time qPCR. An enlarged view of the corresponding ChIP-seq mapping presented in Fig 1 is shown above each ChIP-qPCR plot. Gene promoters are solid boxes and the direction of transcription is shown by arrows. Rabbit IgG was used as negative antibody control and enrichment is not visible in the ChIP-qPCR plots. ***; P<0.001. NS; non-significant.

For further confirmation of the ChIP-seq results, we selected four genes from each of two viral genomic regions, one representing the region of high SUMO-2/3 enrichment with low H3K9me3 mark (orf46, K-bZIP, K8.1 and orf52) (Fig 1, red box) and the other one representing the region of high H3K9me3 mark with little SUMO-2/3 enrichment (orf19, orf20, orf23 and orf25) (Fig 1, blue box) during KSHV reactivation. Consistent with the ChIP-seq results, real-time qPCR data showed that the genes in SUMO-2/3 highly enriched region tested here displayed significant enrichment of SUMO-2/3 after viral reactivation when compared with the non-induced control cells (Fig 2, upper panel). In contrast, the genes in high H3K9me3 mark region showed little increase in SUMO-2/3 modification (Fig 2, lower panel). The reproducibility of this assay was obtained with independent repeat ChIP-qPCR experiment (S2B Fig). Expression of Flag-tagged SUMO-1 and SUMO-2 followed by ChIP using Flag antibody confirmed this phenomena (S3 Fig). These data indicate that the KSHV genome undergoes SUMO-2/3-specific modification following reactivation.

### Correlation of SUMO patterns with active and repressive chromatin marks during KSHV reactivation

The discovery of the negative correlation between SUMO-2/3 and H3K9me3 prompted us to further explore the association of SUMO-1 and SUMO-2/3 with different histone marks. We compared the binding pattern of SUMO-1 and SUMO-2/3 before and after viral reactivation from this study with the previously published binding profiles of various chromatin marks [20] (Table 1). Pearson correlation showed that SUMO-2/3 is medium positive correlated with H3K4me3 (r = 0.3) and medium negative correlated to H3K27me3 (r = -0.3) at 0 hpi. A previous study of global SUMO modification on the human genome also showed that the majority of SUMO paralogs are highly correlated with the active histone mark H3K4me3. Moreover, it was demonstrated that SUMO strongly associated at active promoters, with SUMOylation acting to restrain the gene expression [12]. In line with this study, the positive correlation of SUMO-2/3 with the active histone mark H3K4me3 and its negative correlation of repressive mark H3K27me3 in KSHV latent genomes suggest that SUMO-2/3 may be involved in maintaining a repressive environment in euchromatic regions of the viral episome to restrain viral gene expression during latency. The compelling correlation between SUMO-2/3 and activating histone marks on the KSHV genome prompted us to speculate that SUMO-2/3 may play a role in repressing lytic promoters associated with activating histone marks during latency. To study this, we transiently transduced a lentiviral vector expressing inducible shRNA for SUMO-2/3 into TREx-BCBL-1 [31]. Successful induction of partial SUMO-2/3 knockdown was detected at 24 hours (S4A and S4B Fig). However, SUMO-2/3 knockdown did not induce the expression of viral lytic genes located in the high SUMO-2/3 enrichment region in latent KSHV infected BCBL-1 cells (S4C Fig). This result indicates that knockdown SUMO-2/3 alone is not sufficient to induce KSHV lytic gene expression or viral reactivation from latency.

**Table 1:** Correlation between SUMO paralog occupancy and histone marks in KSHV genome.

	Histone mark	0 hr	12 hr
**SUMO-1**	H3	0.0	-0.1
	H3K9me3	-0.2	-0.2
	H3K27me3	-0.2	-0.2
	AcH3	0.1	0.1
	H3K4me3	0.2	0.2
**SUMO-2/3**	H3	-0.1	0.0
	H3K9me3	-0.2	-0.3
	H3K27me3	-0.3	-0.2
	AcH3	0.2	0.2
	H3K4me3	0.3	0.2

Note: correlation coefficients were calculated according to Pearson.

Abbreviation: H3: Histone 3, H3K9me3: histone H3K9 trimethylation, H3K27me3: histone H3K27 trimethylation, AcH3: acetylated H3, H3K4me3: histone H3K4 trimethylation.

Consistent with our ChIP-on-chip data, SUMO-2/3 showed medium negative correlation to H3K9me3 (r = -0.3) at 12 hpi. Interestingly, no correlation was found between SUMO-2/3 enrichment with any other histone marks after KSHV reactivation. Moreover, no statistically significant correlation was found in SUMO-1 with histone marks on both latent and lytic KSHV genomes. To further explore if the negative correlation of SUMO-2/3 enrichment with heterochromatin mark H3K9me3 is also true on viral promoter regions, a number of promoters with high SUMO-2/3 enrichment at 12 hpi from this study were again compared with the previous study [20]. Among the 36 viral promoters that have high SUMO-2/3 enrichment (4-fold enrichment) during KSHV reactivation, 17 (47%), 15 (42%) and 14 (39%) also contain H3K4me3, AcH3 (H3K9/K14ac), and H3K27me3 marks, respectively. Consistent with results shown in Fig 1 and Table 1, SUMO-2/3 enrichment during viral reactivation is largely devoid of H3K9me3. Only 5 (14%) of the SUMO-2/3 enriched promoters have the H3K9me3 mark. Since H3K9me3 marks are associated with heterochromatin, these results again imply that SUMO-2/3 may be focused on tagging and regulating viral promoters in euchromatin sites during viral reactivation.

### Association of SUMO-2/3 modification and viral gene transcription during KSHV lytic reactivation

**Fig 3:**
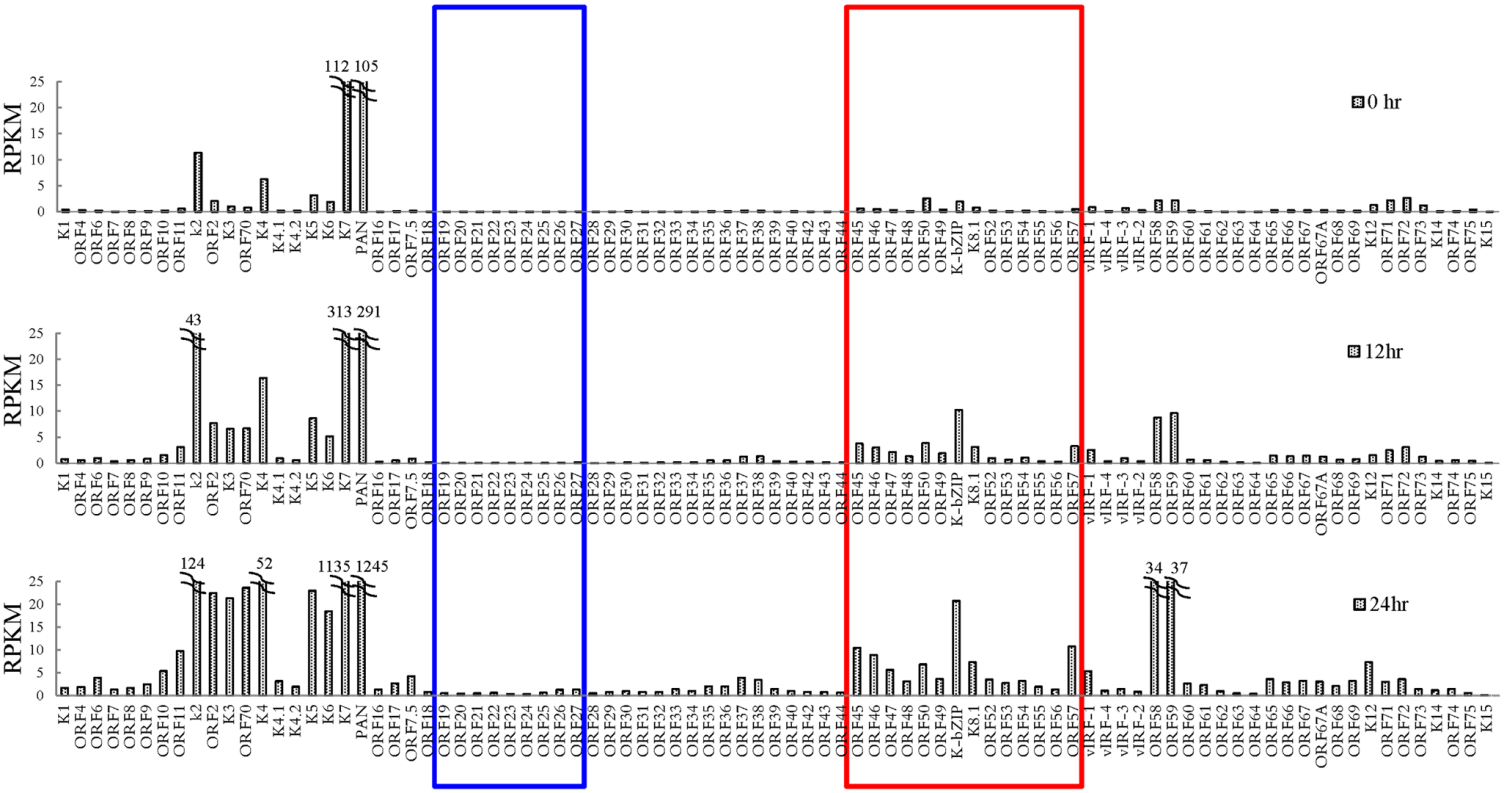
Transcriptome profile of KSHV during viral reactivation. Total RNA samples isolated from non-induced (0 hour) and 0.2 μg/ml Dox-induced (for 12 and 24 hours) TREx-F3H3-K-Rta BCBL-1 cells were subjected to RNA-seq. Expression of all KSHV genes are presented as reads per kilobase per million mapped (RPKM). The rectangular areas are regions that comprise high SUMO-2/3 (red) or high H3K9me3 (blue) levels after KSHV reactivation as in Fig 1.

In order to gain insight into transcriptional regulation by SUMO-2/3 during KSHV reactivation, an RNA-Seq assay was performed using total RNA purified from TREx-F3H3-K-Rta BCBL-1 cells before and after K-Rta induction. Several viral lytic genes, such as K7 and PAN, showed high expression in control cells. This may due to the spontaneous reactivation of KSHV in a small population of BCBL-1 cells. Viral gene expression changes revealed by this assay show that SUMO-2/3 enrichment was preferentially located at gene regions that show a higher level of transactivation during KSHV reactivation (Fig 3, red box). Interestingly, a relatively low level of viral gene expression before and after K-Rta induction was identified in the KSHV genomic region with high H3K9me3 mark (Fig 3, blue box).

**Fig 4:**
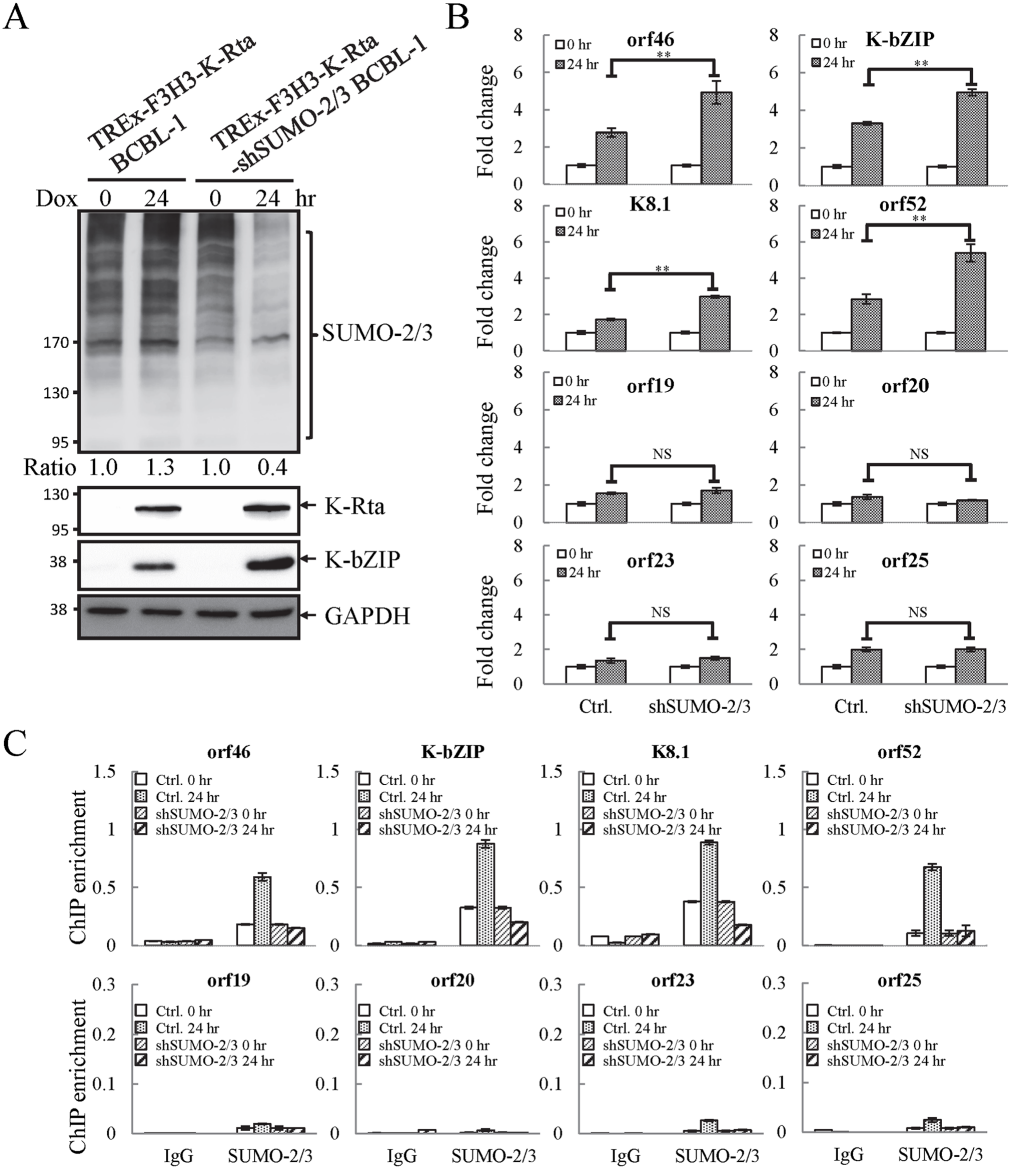
Knockdown of SUMO-2/3 increases transactivation of KSHV lytic genes and reduces SUMO-2/3-specific modification located in the high SUMO-2/3 enrichment region during viral reactivation. (A) TREx-F3H3-K-Rta BCBL-1 and TREx-F3H3-K-Rta-shSUMO-2/3 BCBL-1 cells were treated with Dox (0.2 μg/ml) for 24 hours. Total cell lysates (TCLs) were analyzed by immunoblotting using anti-SUMO-2/3, anti-K-Rta, and anti-K-bZIP antibodies. Anti-GAPDH antibody was used for loading control. Ratio for each cell line is the SUMO-2/3/GAPDH signal observed for Dox treatment at 0 (for shSUMO-2/3) and 24 hour using TREx-F3H3-K-Rta BCBL-1 cells at 0 hour set as 1.0. (B) Total RNA isolated from cells treated as described in (A) was reverse transcribed using oligo-d(T)_18_ primer. The expression level of four viral genes representing SUMO-2/3 enrichment region and four viral genes representing H3K9me3-rich region as described in Fig 1 were quantified by real-time qPCR. All reactions were run in triplicate and normalized against GAPDH. The fold change was computed by comparing induced values to their non-induced controls. ctrl.; control, TREx-F3H3-K-Rta BCBL-1. **; P<0.005. NS; non-significant. (C) ChIP was performed using chromatin prepared from cells treated as described in (A) using anti-SUMO-2/3 antibody. Rabbit IgG was used as negative antibody control and enrichment is not visible in some plots. SUMO-2/3 binding to SUMO-2/3 enrichment and H3K9me3-rich regions were analyzed by real-time qPCR using primer pairs specific for the KSHV loci as described in Fig 2.

As mentioned earlier, the positive correlation between SUMO-2/3 enrichment and viral gene transactivation suggests that SUMOylation on the viral genome may be required for gene shut-off after viral lytic reactivation. To explore this idea, a SUMO-2/3 inducible knockdown TREx-F3H3-K-Rta-shSUMO-2/3 BCBL-1 cell line was used [10]. A time course analysis showed the earliest time point we were able to detect the partial knockdown of SUMO-2/3 was at 24 hours (S5 Fig). Therefore, a 24 hour time point was used for further study. To study the role of SUMO-2/3 in transcriptional regulation, protein and mRNA samples were collected from control (TREx-F3H3-K-Rta BCBL-1) and SUMO-2/3 knockdown TREx-F3H3-K-Rta-shSUMO-2/3 BCBL-1 cells before and after Dox (0.2 μg/ml) treatment for 24 hours. Western blot analysis confirmed the successful induction of K-Rta, expression of K-bZIP and knockdown of SUMO-2/3 at 24 hours after Dox treatment (Fig 4A). The viral gene expression in control (TREx-F3H3-K-Rta BCBL-1) and SUMO-2/3 knockdown TREx-F3H3-K-Rta-shSUMO-2/3 BCBL-1 cells were then compared. Again, the viral genes representing the high SUMO-2/3 enrichment region (orf46, K-bZIP, K8.1 and orf52) and the high H3K9me3 mark region (orf19, orf20, orf23 and orf25) were selected for RT-qPCR study. As shown in Fig 4B, SUMO-2/3 knockdown resulted in a higher induction of the expression of viral genes located in the high SUMO-2/3 enrichment region, but not the genes located in high H3K9me3 mark region. There are also KSHV genomic regions with modest SUMO-2/3 increase that are enriched in H3K9me3, such as the region containing orf34 to orf39 (Fig 1). RT-qPCR results showed that SUMO-2/3 is also essential for restraining the transcription of genes such as orf35, orf37 and orf39 in this region (S6A Fig). This data suggest that SUMO may function at a level higher than histone marks, for example, by recruiting different transcription regulators. However, the latent transcript region containing orf73, orf72, and orf71 that contains both SUMO-1 and SUMO-2/3 enrichment does not show a significant difference in gene induction after SUMO-2/3 knockdown (S6B Fig). This result suggests that the regulatory role of SUMO-2/3 in viral latent gene expression may differ from that of lytic genes. The differential role of SUMO paralogs in regulating KSHV latent genes is of interest and worth further exploration. These results together indicate that SUMOylation enrichment on the viral genome during reactivation is indeed required for diminution of the expression of select viral lytic genes after reactivation.

To confirm whether global SUMO-2/3 knockdown results in a corresponding decline in SUMO-2/3 enrichment on the KSHV genome during reactivation, another ChIP assay was performed using control (TREx-F3H3-K-Rta BCBL-1) and SUMO-2/3 knockdown TREx-F3H3-K-Rta-shSUMO-2/3 BCBL-1 cells. ChIP-qPCR result showed that SUMO-2/3 knockdown abolishes SUMO-2/3 enrichment on the high SUMO-2/3 enrichment region of KSHV genome, but has little effect on the high H3K9me3 mark region (Fig 4C). This result again demonstrates the transcriptional regulation of orf46, K-bZIP, K8.1 and orf52 located in the high SUMO-2/3 enrichment region is dependent on SUMO-2/3. Taken together, these data indicate that KSHV uses SUMO-2/3 modification as an epigenetic modification to dampen lytic gene expression in regions where the H3K9me3 heterochromatin mark is low, in order to modulate transactivation during the lytic cycle. To determine if knockdown SUMO-2/3 led to changes in H3K9me3 mark or additional active and repressive chromatin marks in the KSHV genome, ChIP assays were performed in control (TREx-F3H3-K-Rta BCBL-1) and SUMO-2/3 knockdown TREx-F3H3-K-Rta-shSUMO-2/3 BCBL-1 cells using antibodies specific for different histone marks. Knockdown of SUMO-2/3 did not change any of the interrogated histone marks in the KSHV genome (S7 Fig). Consistent with our previous result (S6A Fig), these data indicate that SUMOylation may modify protein binding at a level above the deposition of specific histone marks.

### Knockdown of SUMO-2/3 enhances KSHV virion production

**Fig 5:**
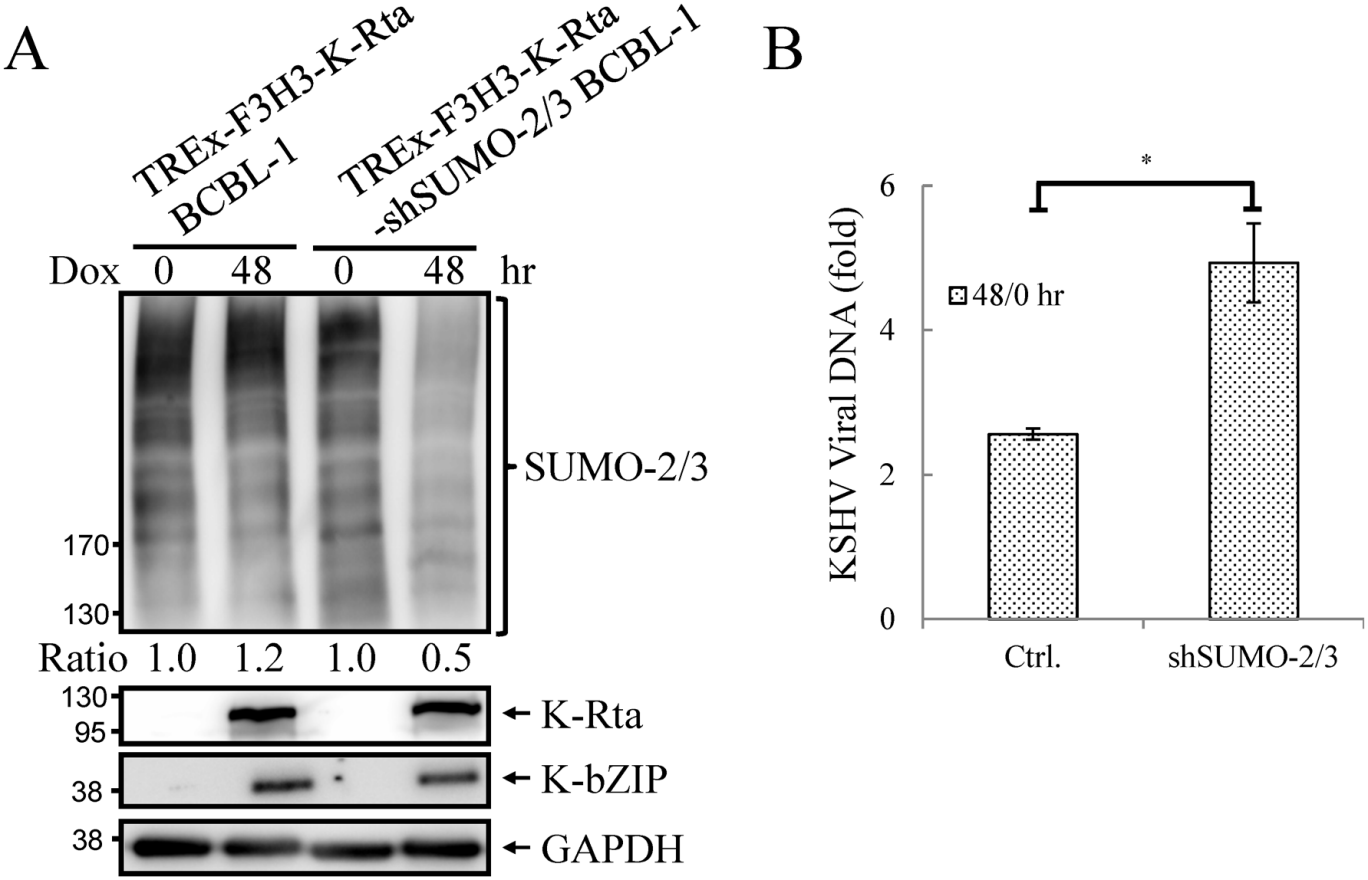
Knockdown SUMO-2/3 enhances KSHV virus production. (A) TCLs from non-induced (0 hour) and 0.2 μg/ml Dox-treated (48 hours) TREx-F3H3-K-Rta BCBL-1 and TREx-F3H3-K-Rta-shSUMO-2/3 BCBL-1 cells were analyzed by immunoblotting using antibodies as described in Fig 4A. Ratio for each cell line is the SUMO-2/3/GAPDH signal observed for Dox treatment at 0 (for shSUMO-2/3) and 48 hour using TREx-F3H3-K-Rta BCBL-1 cells at 0 hour set as 1.0. (B) Supernatants from TREx-F3H3-K-Rta BCBL-1 and SUMO-2/3 knockdown TREx-F3H3-K-Rta-shSUMO-2/3 BCBL-1 cells were collected and filtered at 0 and 48 hours after Dox (0.2 μg/ml) treatment. Virion-associated DNA was purified and KSHV DNA levels were determined by TaqMan qPCR. Mean ± SD. Fold was determined by KSHV DNA copy number/μl at 48 hour divided by KSHV DNA copy number/μl at 0 hour. ctrl.; control, TREx-F3H3-K-Rta BCBL-1. *; P<0.05.

Enhancement of viral lytic gene expression by SUMO-2/3 knockdown may lead to an increase in viral replication and elevate levels of virus production. To examine the level of KSHV virion production in SUMO-2/3 knockdown cells, the control (TREx-F3H3-K-Rta BCBL-1) and the inducible SUMO-2/3 knockdown TREx-F3H3-K-Rta-shSUMO-2/3 BCBL-1 cells were treated with 0.2 μg/ml Dox for 48 hours, a condition that we can consistently detect relatively low (~ 2- to 3-fold virion induction) but significant induction of KSHV virion production in control TREx-F3H3-K-Rta BCBL-1 cells. The successful induction of K-Rta, expression of K-bZIP and knockdown of SUMO-2/3 was first assessed by immunoblotting (Fig 5A). The supernatants collected from different treatments were used for virion purification and the level of virion-associated DNA was determined using real-time TaqMan qPCR amplification. Consistent with our prediction, SUMO-2/3 knockdown significantly increased viral production by ~2-fold over control cells (Fig 5B). Together, these data indicate that SUMO-2/3 modification may specifically target the KSHV genome to create a silencing chromatin environment ready for diminution of lytic gene expression and viral replication after induction.

### Generation of recombinant KSHV BACmid with E3 ligase-dead mutant of K-bZIP (K-bZIP-L75A)

**Fig 6:**
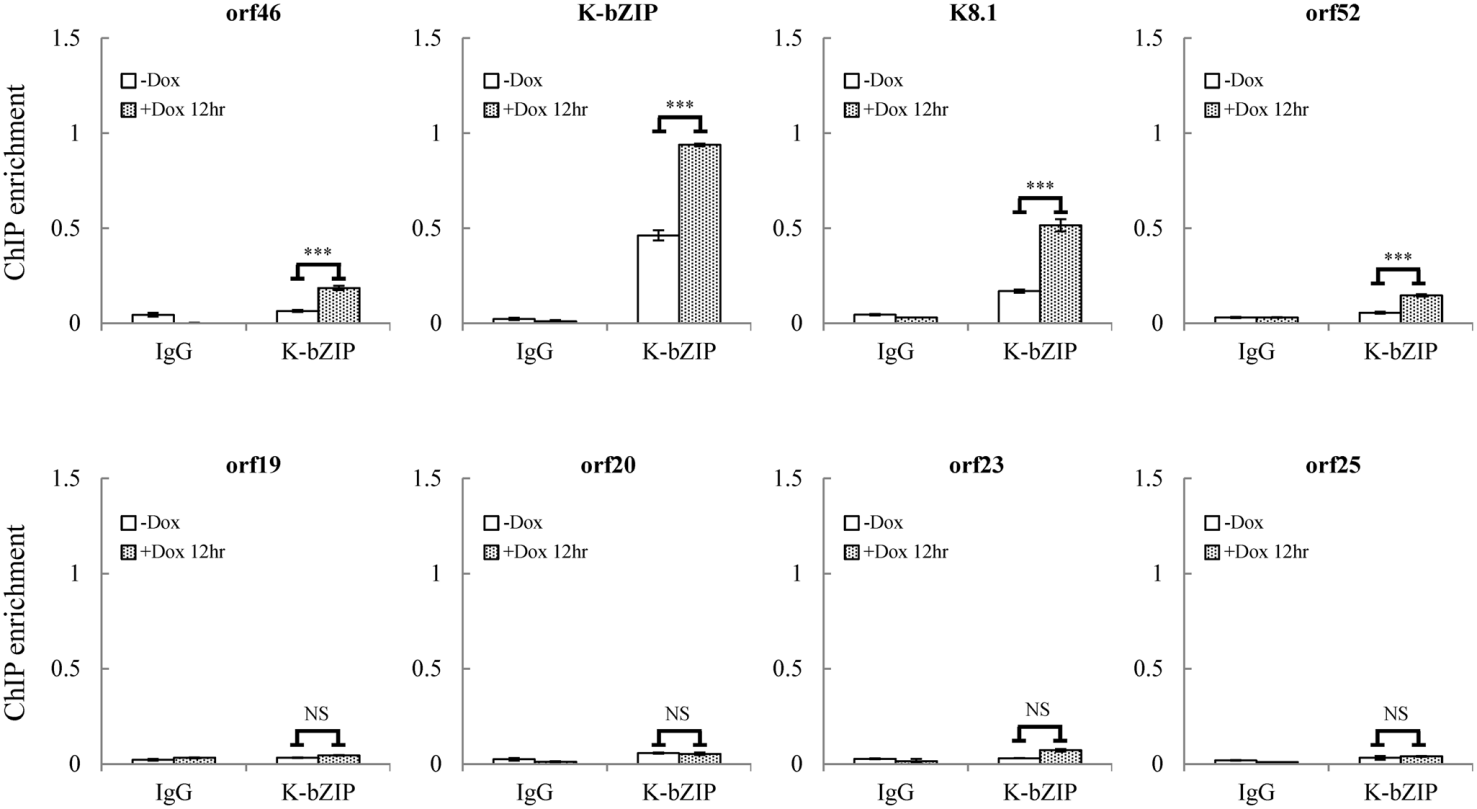
K-bZIP binding on selected KSHV promoters in TREx-F3H3-K-Rta BCBL-1 cells. ChIP was performed using chromatin prepared from cells treated as described in Fig 1 using anti-K-bZIP antibody. Rabbit IgG was used as negative antibody control. K-bZIP binding to orf46, K-bZIP, K8.1, and orf52 promoters in the SUMO-2/3 enriched region and orf19, orf20, orf23, and orf25 promoters in H3K9me3-rich region were analyzed by real-time qPCR.

Our findings strongly suggest that SUMO-2/3 may play a major and critical role in regulating viral gene silencing after transactivation. The next question is how KSHV controls the SUMO-2/3 modification on its genome during lytic reactivation. Interestingly, our recent report demonstrated a unique mechanism by which KSHV modulates SUMOylation via expressing a viral lytic SUMO E3 ligase, K-bZIP [27]. This viral SUMO E3 ligase has specificity towards SUMO-2/3. Together with this finding, we speculated that during KSHV reactivation, KSHV expresses the lytic protein K-bZIP and simultaneously conjugates SUMO-2/3 to viral genome regions with low heterochromatin marks. To address this, a ChIP assay was performed using a K-bZIP specific antibody and chromatin prepared from TREx-F3H3-K-Rta BCBL-1 cells before and after Dox induction for 12 hours. Again, primer pairs for orf46, K-bZIP, K8.1 and orf52 representing the high SUMO-2/3 enrichment region and orf19, orf20, orf23 and orf25 representing the high H3K9me3 mark region were used to determine the chromatin binding of K-bZIP using real-time qPCR. Consistent with our hypothesis, a significant higher increase of K-bZIP binding in SUMO-2/3 enrichment region after KSHV reactivation was observed (Fig 6). Note that some K-bZIP binding on its own promoter was observed in non-induced cells. ChIP data reported in Ellison *et al*. 2009 stated that the K-bZIP promoter was the most enriched target of K-bZIP following its overexpression [32]. Thus, the interaction between K-bZIP and its promoter may be high and readily detectable when compared with other KSHV promoters. A small population of BCBL-1 cells continuously emerge from latency into lytic replication resulting in the expression of K-bZIP in a small fraction of cells, thus some binding of K-bZIP to its promoter is observed in control cells. Together, these results suggest that K-bZIP may be the SUMO E3 ligase catalyzing the addition of SUMO-2/3 to the KSHV genome during reactivation.

**Fig 7:**
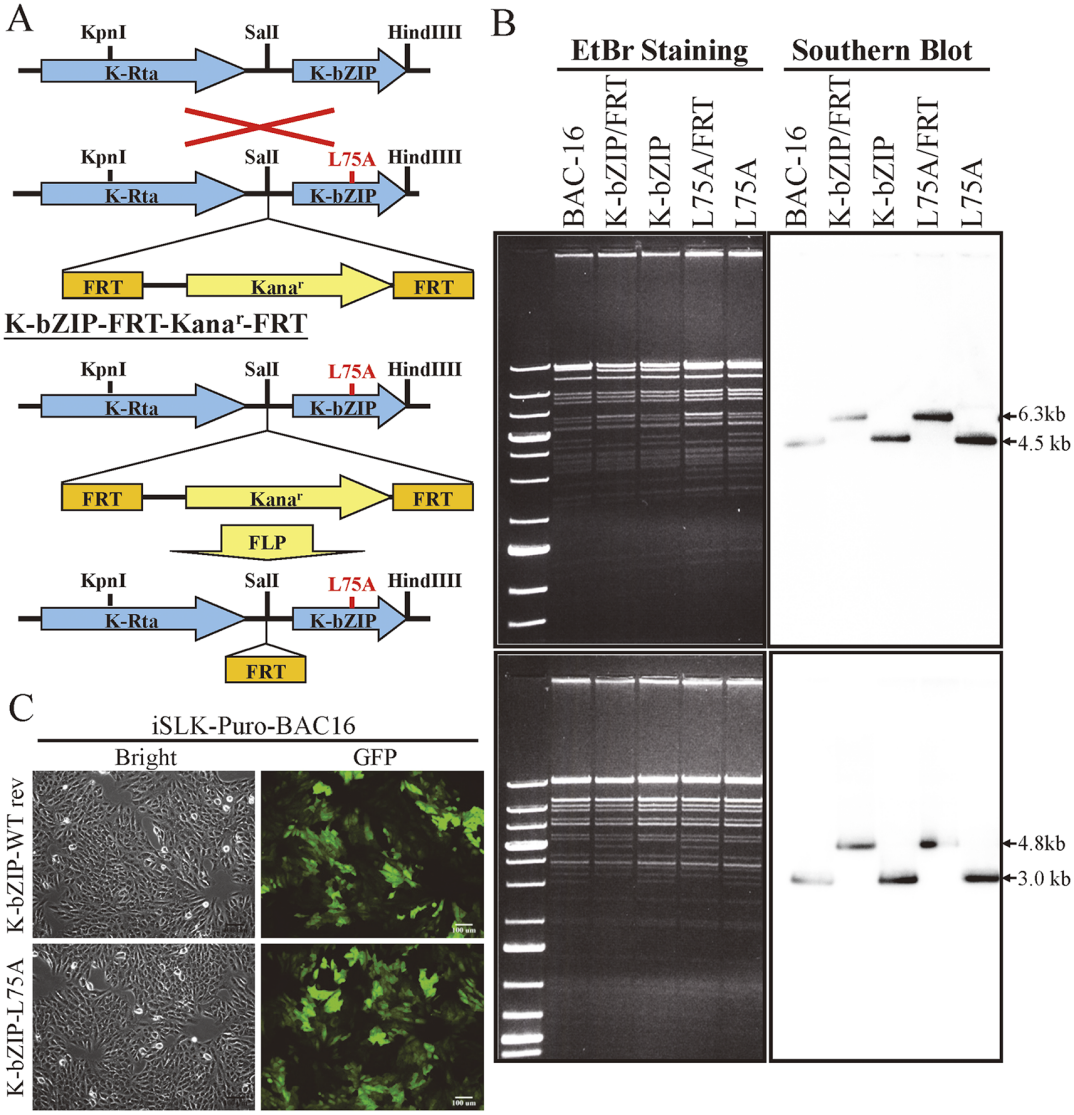
Generation of K-bZIP-L75A mutated and wild-type revertant (WT rev) recombinant KSHV (rKSHV) in BAC16. (A) Schematic representation of the recombineering procedures used for construction of K-bZIP-L75A mutants in BAC16. Recombination between the KSHV genomic locus (top) and the K-bZIP L75A targeting vector (bottom) is illustrated in the upper part of the figure. FLP recombinase-mediated removal of the targeting cassette is depicted in the lower portion. (B) Agarose gel and Southern blot analysis of recombineered BAC16 that was digested with KpnI (upper panel, left) or HindIII (lower panel, left) and probed with a radiolabeled K-bZIP DNA fragment (right). (C) Stable cell line of iSLK-Puro-BAC16 shows >90% GFP-positive cells. Phase (left) and FITC channel (right) images are shown.

To uncover the potential functional role of the SUMO E3 ligase activity of K-bZIP in regulating KSHV gene expression during reactivation in a virus context, we generated a SUMO E3 ligase dead mutant of K-bZIP in BAC16, a KSHV BAC clone generated from Dr. Jung’s laboratory [33]. The leucine 75 (L75) in the SIM domain of K-bZIP was mutated to alanine in the targeting vector using site-directed mutagenesis [27]. This K-bZIP-L75A mutant was introduced into the wild-type (WT) KSHV genome in BAC16 bacmid by recombineering (Fig 7A). Recombination was carried out using a SW105 transformant containing BAC16. A targeting vector containing WT K-bZIP was also used to replace the K-bZIP-L75A allele on BAC16 to generate revertant viruses for use as WT control (WT rev). The BAC16 constructs were first checked by PCR analysis. The positive clones were then digested with KpnI or HindIII and analyzed by agarose gel electrophoresis (Fig 7B; left panel) and subsequently by Southern blot analysis (Fig 7B; right panel). Both KpnI and HindIII digestion analysis shows a ~1.8 Kb band shift in BAC16 intermediate and return to similar position of BAC16 parental band upon FRT-mediated removal of kanamycin selection cassette (Fig 7B). Sequencing was used to confirm successful mutagenesis and no unexpected changes were detected. After confirmation, WT rev and L75A mutant viruses were reconstituted in iSLK cells, a cell line that inducibly expresses K-Rta by Dox treatment. Following hygromycin selection for 21 days, comparable GFP expression was observed in iSLK-Puro-BAC16 cells (Fig 7C). The expression of KSHV latent protein LANA was analyzed by immunoblotting, indicating stable propagation of BAC16 in mammalian cells (S8A Fig). The KSHV genome copy number in stable iSLK-Puro-BAC16 K-bZIP-WT rev and -L75A mutant cell lines was analyzed by qPCR using orf19 and orf20-specific primer pairs. Similar relative copy numbers of KSHV genome were observed from both primer pairs (S8B Fig) and the values were used to normalize all the following real-time RT-qPCR and virus production quantification experiments.

### K-bZIP, a viral SUMO E3 ligase, regulates viral gene expression and reactivation

**Fig 8:**
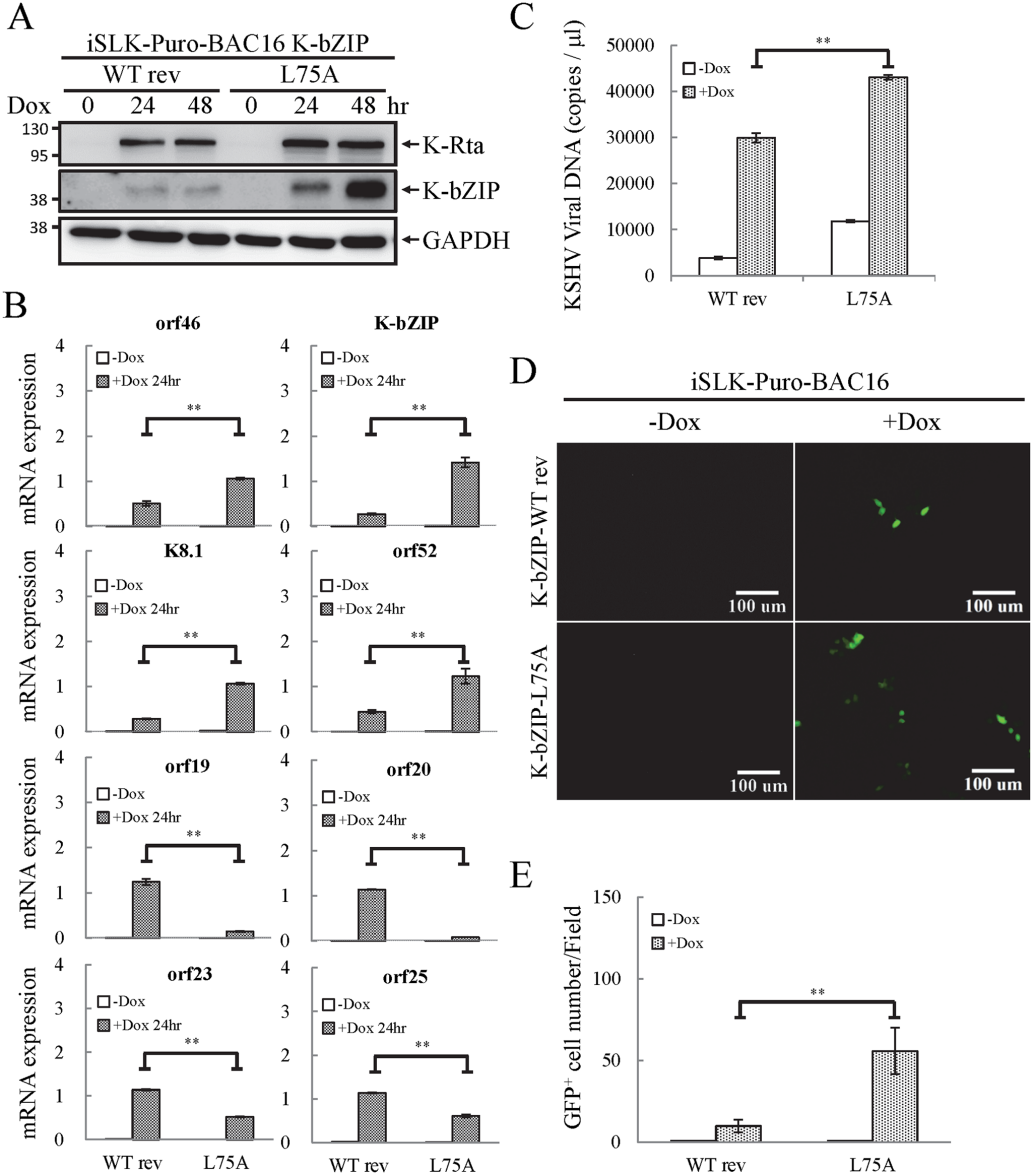
The SUMO E3 ligase activity of K-bZIP is necessary to alleviate the increase in gene expression and virus production during reactivation. (A) TCLs were collected from non-induced (0 hour) and 1 μg/ml Dox treated (for 24 and 48 hours) iSLK-Puro-BAC16 K-bZIP-WT rev and -L75A cell lines. Immunoblotting was used to confirm the induction of K-Rta and expression of K-bZIP. GAPDH was used as loading control. (B) Total RNA isolated from non-induced (0 hour) and 1 μg/ml Dox treated (24 hours) iSLK-Puro-BAC16 K-bZIP-WT rev and -L75A cells was reverse transcribed using oligo-d(T)_18_ primer and analyzed by real-time qPCR using specific primer pairs representing genes in SUMO-2/3 enrichment and H3K9me3-rich regions. All reactions were run in triplicate and normalized against GAPDH. (C) Supernatants from iSLK-Puro-BAC16 K-bZIP-WT rev and -L75A cells were collected at 0 and 48 hours after Dox (1 μg/ml) treatment and filtered. Virion-associated DNA was purified and KSHV DNA levels were determined by TaqMan qPCR. Mean ± SD. **; P<0.005. (D) 293T cells were infected with filtered supernatants harvested from iSLK-Puro-BAC16 K-bZIP-WT rev and -L75A cells treated with or without 1 μg/ml Dox for 72 hours. GFP positive cells were analyzed by fluorescence microscopy (FITC, 10X magnification) 48 hours after infection. (E) The GFP positive cells were quantified using the average from >20 microscopic fields. Mean ± SD. **; P<0.005.

To evaluate the potential role of K-bZIP SUMO E3 ligase, iSLK cells harboring different recombinant KSHV-BACmids were induced with Dox for 24 and 48 hours for gene expression and virus production analysis, respectively. Total protein lysates were collected for immunoblotting analysis to assess the successful induction of K-Rta and expression of K-bZIP (Fig 8A). Next, we measured the accumulation of KSHV orf46, K-bZIP, K8.1 and orf52 mRNA, representing the potential SUMO-2/3-regulated genes and orf19, orf20, orf23 and orf25 mRNA, representing non-SUMO-2/3-regulated genes. A significant higher increase in expression of the SUMO-2/3-regulated viral genes was found in iSLK-Puro-BAC16 K-ZIP-L75A mutant compared with K-bZIP-WT rev (Fig 8B; upper panel). Consistent with the significant increase of K-bZIP transcript by approximately 5-fold in iSLK-Puro-BAC16 K-ZIP-L75A mutant, a higher level of mutant K-bZIP protein was also observed during K-Rta-induced viral reactivation (Fig 8A). In line with our previous data, this result indicates that K-bZIP may mediate the SUMOylation of viral promoters in the low H3K9me3 region which results in a diminution of viral gene expression after reactivation. Unexpectedly, viral genes in the high H3K9me3 mark region showed defects in transactivation in the iSLK-Puro-BAC16 K-bZIP-L75A mutant (Fig 8B; lower panel). Since no SUMO enrichment was identified in the high H3K9me3 region, SUMO E3 ligase activity of K-bZIP should not influence the transactivation of genes in this region. As shown in our previous report, a high affinity direct interaction between K-bZIP and H3K9me3 was found [17]. The loss of gene transactivation in the high H3K9me3 region early after reactivation (24 hours) may relate to the direct binding of K-bZIP to this region and enhance late gene expression by recruiting SUMOylated transcription factors in a SIM-dependent manner. It should also be noted that K-bZIP is known to be required for lytic DNA replication [34–36], and that DNA replication induces high level expression of late genes, including orf19, orf20, orf23 and orf25. The SIM domain of K-bZIP may be required for KSHV replication and that is why the SIM mutation results in less expression of orf19, orf20, orf23 and orf25.

To determine if the K-bZIP SIM plays a role in its binding on the viral genome, ChIP assays of K-bZIP by using chromatin prepared from non-induced and Dox-induced iSLK-Puro-BAC16 K-bZIP-WT rev and -L75A mutant were performed. Consistent with our ChIP result of K-bZIP occupancy from TREx-F3H3-K-Rta BCBL-1 cells (Fig 6), a significant higher increase in K-bZIP binding in SUMO-2/3 enrichment region compared with high H3K9me3 mark region was also observed (S9 Fig). However, the binding of K-bZIP to viral promoters shows no significant differences between the WT rev and L75A mutant after KSHV reactivation (S9 Fig). This result suggests that the K-bZIP SIM is not involved in K-bZIP binding on the KSHV genome but may influence its recruitment of other SUMOylated proteins.

### E3 ligase activity of K-bZIP is essential for diminution of KSHV virion production

**Fig 9:**
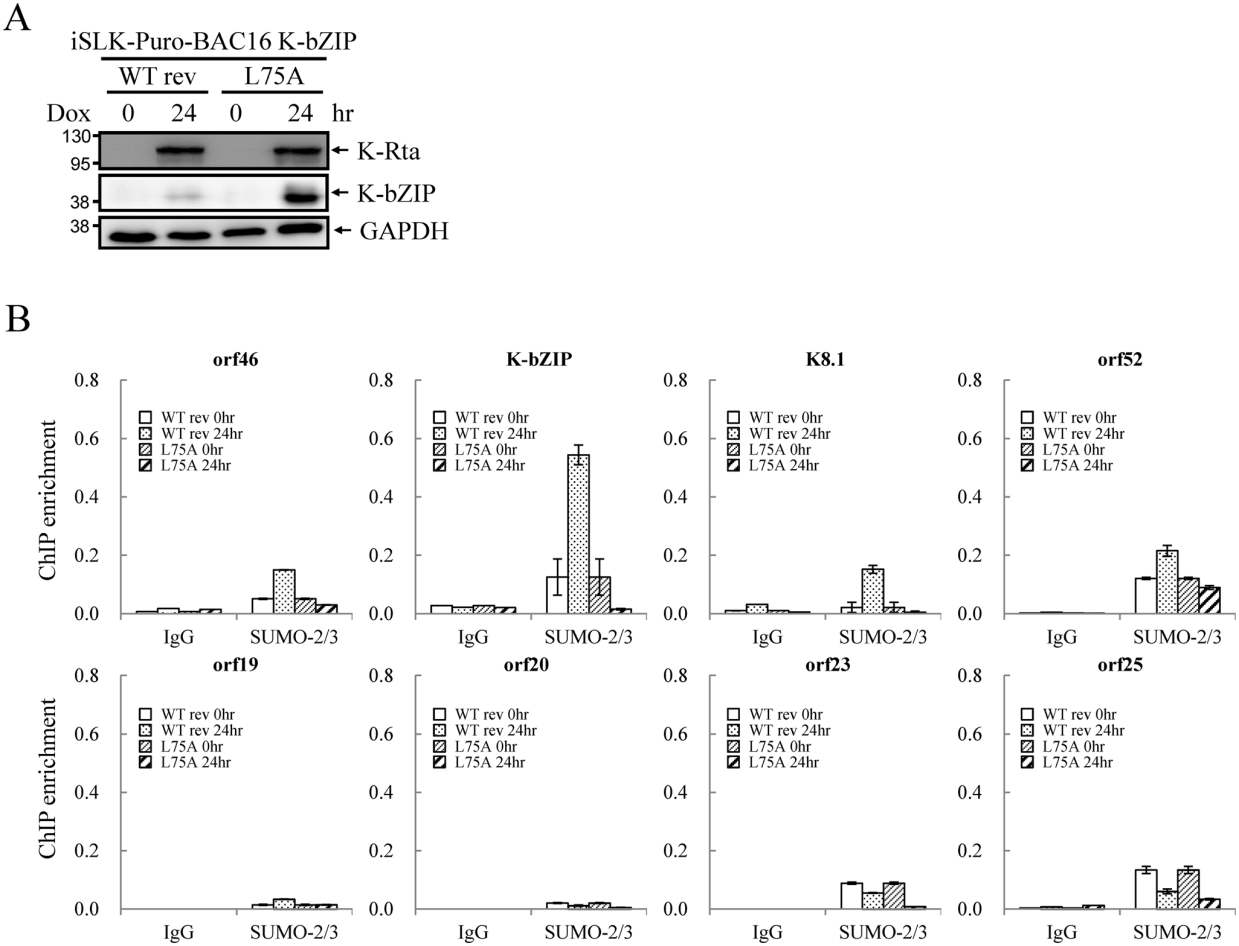
SUMO E3 ligase activity of K-bZIP is essential for SUMO-2/3 modification of the viral genome during lytic reactivation. (A) TCLs were collected from non-induced (0 hour) and 1 μg/ml Dox treated (for 24 hours) iSLK-Puro-BAC16 K-bZIP-WT rev and -L75A cell lines. Immunoblotting was used to confirm the induction of K-Rta and expression of K-bZIP. GAPDH was used as loading control. (B) ChIP was performed using chromatin prepared from cells treated as described in (A) using anti-SUMO-2/3 antibody. The SUMO-2/3 modification on selected promoters in SUMO-2/3 enrichment and H3K9me3-rich regions were analyzed by real-time qPCR. Rabbit IgG was used as negative antibody control and enrichment is not visible in some plots.

Since KSHV lytic reactivation is accompanied by transcriptional reprogramming, we extended the observations to virus production. Supernatants from non-induced and Dox-induced (48 or 72 hours) iSLK-Puro-BAC16 K-bZIP-WT rev and -L75A cells were collected and used for detection of virion-associated DNA using real-time TaqMan qPCR amplification or to infect 293T cells, respectively. In agreement with our previous findings, K-bZIP-L75A mutant showed significantly higher viral production (Fig 8C). Consistently, K-bZIP-L75A mutant also exhibited higher numbers of recombinant KSHV (rKSHV) infected 293T cells by ~3-fold over than WT rev KSHV (Fig 8D and 8E). In addition, when examined on a single-cell basis, K-Rta-expressing cells show more lytic protein expression in iSLK-Puro-BAC16 K-bZIP-L75A cells compared to WT rev during reactivation (S10 Fig). To confirm the higher viral production in iSLK-Puro-BAC16 K-bZIP-L75A cells, the infection experiment was repeated by including the original BAC16 construct. The parental BAC16 shows similar virus production as K-bZIP-WT rev (S11 Fig). K-bZIP-L75A mutant showed higher virus production compared to both parental and WT rev KSHV (S11 Fig). The data from SUMO-2/3 knockdown and K-bZIP SUMO E3 ligase-dead mutant strongly suggest that SUMO E3 ligase activity of K-bZIP is responsible for the SUMO-2/3 enrichment on the KSHV genome during viral reactivation. To confirm this hypothesis, another ChIP assay was performed with chromatin prepared from iSLK-Puro-BAC16 K-bZIP-WT rev and -L75A mutant using anti-SUMO-2/3 antibody. Consistent with all data above, a significant increase of SUMO-2/3 was observed on the promoters of orf46, K-bZIP, K8.1 and orf52 but not of orf19, orf20, orf23, and orf25. Moreover, the SUMO-2/3 enrichment on orf46, K-bZIP, K8.1 and orf52 promoters was completely abolished in K-bZIP SUMO E3 ligase-dead mutant (Fig 9). Consistent with Fig 8A, a higher level of K-bZIP protein was observed during K-Rta-induced viral reactivation (Fig 9A). In line with our data of SUMO-2/3 knockdown (S7 Fig) showing that SUMO modification is involved in gene transcription regulation without changing histone marks, K-bZIP SUMO E3 ligase-dead mutant did not change the H3K9me3 pattern (S12 Fig). Together, these data show that conjugation of SUMO-2/3 on the KSHV genome by the viral SUMO E3 ligase K-bZIP plays an essential role in alleviating transactivation of viral genes and production of virus.

## Discussion

One distinct feature of herpesviruses is that there are two phases, latent and lytic, in their lifecycle. Establishment of latency is a common property for herpesvirus to evade host immune responses and establish life-long infection. In addition to viral propagation, lytic reactivation has also been found to be required for maintaining herpesviral persistence [37]. This is substantiated by mouse experiments showing that passive transfer of anti-lytic cycle antibody, but not anti-latent cycle antibody, into B-cell deficient mice decreased the number of cells harboring latent virus [37]. This idea is further supported by the finding that the antiviral drug cidofavir reduces the frequency of latently infected cells [38]. For KSHV, both latent and lytic cycles are essential for not only its persistent infection but also for its tumorigenesis (reviewed in [39,40]). Therefore, maintaining an exquisite balance between latency-to-lytic cycle switch is important for persistent viral infection. The SUMO-2/3 enrichment identified here during viral reactivation demonstrates an unexpected SUMO function in the diminution of active KSHV lytic gene expression. Although the benefit of KSHV suppressing itself during reactivation is unclear, single cell analysis of cells undergoing reactivation have noted that only 20% of K-Rta positive cells also expressed the late gene K8.1 suggesting the existence of additional commitment factors required for K-Rta positive cells to advance through complete reactivation [41]. Although K-Rta is necessary and sufficient for lytic reactivation, it has been considered an inefficient reactivating switch, subject to positive and negative regulation by viral and cellular factors [42]. Thus, there is precedent for cells putatively undergoing reactivation (i.e., K-Rta positive) to enter sub-lytic, abortive or full lytic pathways. Thus, we speculate SUMO-2/3 deposition may influence viral pathway fate by effects on other viral gene products feeding back to the level of K-Rta. Alternatively, as posited by studies in yeast [5], SUMOylation may function in promoter clearance after each round of activated transcription, allowing another cycle of transcription to proceed if sufficient activator signal is present. Our results (Figs 4B, 4C, 8B and 9B) are consistent with this mechanism; failure to efficiently clear a promoter, through SUMO-2/3 knockdown or K-bZIP mutation would be expected to result in prolonged transcriptional activation and elevated viral reactivation. Lack of SUMO-2/3 enrichment at high H3K9me3 regions (Fig 1) would account for the differential promoter responses observed. Though disruption the balance of latency-to-lytic cycle toward lytic activation might lead to increased infectivity and viral loads, it may also result in host immune activation and viral clearance. Moreover, current anti-herpes viral drugs only target lytic replicating viruses. This concept has been explored as a potential therapy for herpesvirus. Treatment strategies consisting of lytic induction of Epstein–Barr virus (EBV) using doxorubicin and gemcitabine, or both EBV and KSHV by bortezomib, followed with chemo- or radiotherapy has been described [43,44]. In addition, the report of KSHV reactivation following knockdown of Tousled-like kinases [45] suggests that development of small molecule inhibitors targeting these kinases may act as lytic inducers. A somewhat similar approach (the so-called “shock and kill” strategy) that shock latent proviruses with pharmacological agents such as histone deacetylase (HDAC) inhibitors and kill emergent viruses with combined anti-retroviral therapy and/or host cytolytic T cells is currently under evaluation as a means to eradicate latent proviruses present in patient HIV reservoirs (reviewed in [46]). An inhibitor of K-bZIP E3 ligase activity might function similar to L75A and increase lytic replication that favors host clearance by making KSHV visible to the immune system and anti-viral drugs. Understanding the molecular mechanisms that regulate the KSHV latent-to-lytic switch not only holds the key to developing effective therapy for KSHV but also for other oncogenic herpesvirus.

In latent phase, KSHV genome persists as a transcriptionally silent extrachromosomal episome resembling heterochromatin. During lytic phase, many regions of the viral genome adopt a state of euchromatin organization and almost all viral genes are transcribed in a temporally ordered manner. Epigenetic modifications of herpesvirus chromatin very likely play key roles in regulation viral gene expression as well as controlling the switch between latency and lytic replication. This notion is supported by the fact that viral lytic reactivation can be induced by inhibitors of DNA methyltransferases (DNMTs) [47] and HDACs [48,49]. Several recent studies comprehensively analyzed the epigenetic marks, including DNA methylation and histone modifications, in the KSHV latent and lytic genome [18–20]. Upon *de novo* infection, a quick transition from euchromatin mark to heterochromatin mark was detected in KSHV genomes [19]. In latent KSHV genomes, both activating as well as repressive histone marks were identified at certain viral loci. This “bivalent” state of chromatin generated a poised state of repression that can likely be quickly reverted to fully active state upon induction of viral lytic cycle under stimuli [18]. During viral reactivation, the activating marks located on genomic regions encoding the immediate-early (IE) genes were increased whereas the repressive H3K27me3 mark was decreased [20]. Together, these results highlight the importance of epigenetic modifications in the regulation of the KSHV lifecycle. Although significant effort has been devoted to find key epigenetic marks and their roles in the establishment of KSHV latent and lytic chromatin, a detailed understanding of the post-translational modifications involved in mediating the switch between KSHV latent infection and lytic replication is still largely unknown.

SUMO modification is a post-translational modification that not only modulates the function of many transcription factors but also the chromatin organization by recruiting chromatin remodeling enzymes, including DNA and histone modification enzymes, to regulate gene expression. Global analysis of SUMOylation in epigenetic regulation has been done in different eukaryotic cells and indicates that SUMOylation can either restrain [10,12] or promote [11] the expression of actively transcribed genes under different environmental stimuli. These results indicate that depending on the stimuli, SUMO paralogs are able to create repressive or active chromatin states to regulate host gene expression. It comes as no surprise that herpesvirus KSHV has evolved ways to modulate the SUMO machinery to epigenetically regulate viral chromatin to benefit its lifecycle. Indeed, our recent report showed that KSHV encodes a lytic SUMO-2/3 specific E3 ligase, K-bZIP [27]. Moreover, Cai *et al* showed that the KSHV latent protein LANA also contains a SUMO-2 specific SIM that is essential for the recruitment of the SUMOylated chromatin remodeling protein KAP-1 which aids in the maintenance of viral latency [50]. These studies prompted us to study how SUMOylation regulates viral gene expression during reactivation and whether there is preferential SUMO paralog usage. ChIP-seq results showed similar global SUMO-1 and SUMO-2/3 binding patterns on KSHV latent, but not lytic, viral genomes. SUMO-2/3, compared with SUMO-1, was significantly increased across the KSHV genome during reactivation (Fig 1). The significant enrichment of SUMO-2/3 on the viral genome provided us with an opportunity to uncover the epigenetic role of SUMO-2/3. The role of SUMO-2/3 in regulating viral gene expression during KSHV reactivation was revealed by RNA-seq conducted in TREx-F3H3-K-Rta BCBL-1 cells before and after KSHV reactivation (Fig 3). The higher induction of viral gene expression in high SUMO-2/3 enriched regions during viral reactivation allows for two potential hypothesis; (1) SUMO-2/3 activates viral gene transcription during reactivation and (2) SUMO-2/3 restrains viral gene expression after reactivation. To elucidate the functional role of SUMO modification in transcription regulation, SUMO-2/3 knockdown experiments were conducted. Reduction of SUMO-2/3 binding at a high SUMO-2/3 enrichment region on the KSHV genome was first confirmed in SUMO-2/3 knockdown TREx-F3H3-K-Rta-shSUMO-2/3 BCBL-1 cells (Fig 4C). During reactivation, the expression of viral genes in the high SUMO-2/3 enrichment region was activated to a higher level relative to a low SUMO-2/3 enrichment region after SUMO-2/3 knockdown (Fig 4B). Together with previous reports [10–12], these results indicate the preferential usage of SUMO-2/3 by KSHV in restraining viral gene expression during reactivation.

Histone marks have long been studied and believed to be essential in maintaining chromatin structure and therefore manipulating gene expression. Though global SUMO modifications in epigenetic regulation have begun to be studied in eukaryotic cells, the correlation between histone marks and SUMO modifications has not yet been elucidated. Due to the relatively low complexity of the viral genome and the availability of genome-wide histone modification landscapes from KSHV, we were able to compare the genome-wide distribution of SUMO modification with different histone marks [20] on KSHV latent and lytic genomes. Our SUMO DNA binding patterns were overlaid with different epigenetic marks on the KSHV genome. Correlation with histone marks was only identified in chromatin binding of SUMO-2/3, but not SUMO-1 (Table 1). An interesting finding is that our ChIP-seq data showed that during lytic reactivation, SUMO-2/3 levels increased mostly on KSHV genomic regions devoid of H3K9me3, thus negatively correlating with H3K9me3 occupied late gene-rich regions (Fig 1). The genome-wide correlation analysis also showed that SUMO-2/3 enrichment only correlated with H3K9me3 (r = -0.3) but not any other histone marks. This finding suggests that SUMO modification may be involved in transcriptional regulation of genes at low heterochromatin loci. However, there are also few regions with increased SUMO-2/3 as well as H3K9me3 modifications. Our preliminary data showed that SUMO-2/3 also restrained the transcription of genes in these regions (S6A Fig). This data suggest that SUMO may function at a level higher than histone marks. The regulation of gene expression in those regions may be more complex and worthwhile for detailed analysis in the future.

A previous report from Toth *et al*. noted that the repressive H3K9me3 mark was restricted to two regions of the KSHV genome that mainly encode late viral genes [20]. The absence of SUMO-2/3 enrichment in these two regions indicates that SUMO-2/3 may be less involved in regulating transcription of viral lytic late genes located in heterochromatin regions than early expressed viral lytic genes in euchromatin regions. This idea is further supported by a recent study analyzing the genome-wide SUMOylation sites in human cells. Little SUMO-1 and SUMO-2/3 was found at repressive chromatin regions marked by H3K9me3 [12]. The significant enrichment of SUMO-2/3 in low H3K9me3 region (Fig 1) in conjunction with the higher transcription activation of genes in this region after SUMO-2/3 knockdown (Fig 4B) suggests a novel level of interpretation that, in order to attenuate reactivation, expression of immediate early and early gene located in open chromatin regions may be repressed by SUMO-2/3 modification upon stimulation for viral reactivation. These results are consistent with the study in yeast suggesting that SUMO functions in the shut-off of induced genes after stimuli are no longer present [5]. However, we cannot completely exclude the possibility that SUMO-2/3 may participate in transcriptional activation of some viral genes during viral reactivation as one recent global SUMO-1 study mentions that SUMO-1 modification is responsible for stimulation of promoter activity [11]. Together, these findings illustrate the complexity of SUMOylation-mediated epigenetic regulation of transcription under different environmental conditions.

SUMO E3 ligases occasionally display paralog-specificity toward certain targets, however, none of the cellular SUMO E3 ligases, including protein inhibitor of activated STAT (PIAS) family, Ran-binding protein 2 (RanBP2), and Pc2, have been demonstrated to have selectivity towards a specific SUMO paralog. Therefore, despite many studies intent on analyzing paralog-specific SUMO modifications of epigenetic and transcription regulators, the distinctive functional specificity of SUMO isoforms in global epigenetic regulation in relationship to gene expression remains largely unknown. This indicates that a specific stimulus that can induce SUMO paralog-specific modification is required to elucidate the SUMO paralog-specific epigenetic regulatory function. By encoding a viral lytic SUMO-2/3 specific E3 ligase, K-bZIP, KSHV possesses as an ideal tool to distinguish the functional specificity of SUMO-2/3 in epigenetic regulation [27]. To study the functional role of SUMO-2/3 specific E3 ligase activity in KSHV gene expression, we generated a SUMO E3 ligase-dead mutant of K-bZIP (K-bZIP-L75A) in the context of the KSHV genome. Analyses of cells containing the K-bZIP-L75A mutant bacmid during KSHV reactivation demonstrated a repressive role of K-bZIP SUMO-2/3 specific E3 ligase activity in regulating the expression of viral genes located in a high SUMO-2/3 enrichment genome region (Fig 8B). This repressive function of K-bZIP helped to dampen KSHV viral production upon reactivation stimuli (Fig 8C and 8E). Moreover, our result demonstrated that E3 ligase activity of K-bZIP is indeed responsible for SUMO-2/3 enrichment on euchromatin regions of the KSHV genome during reactivation (Fig 9B). These results suggest that K-bZIP may mediate the SUMOylation of DNA or histone binding proteins located in KSHV genome euchromatin regions and alleviate transactivation during viral reactivation.

The identity of the protein(s) modified by SUMO and responsible for global SUMO-2/3 enrichment on the viral genome during reactivation is an important unanswered question. All K-bZIP interacting chromatin binding proteins could be potential candidates, such as the H3K9me3 demethylase JMJD2A [17]. Another potential SUMO-2/3 target might be K-bZIP itself. K-bZIP is a SUMO-2/3 specific E3 ligase and residue Lys158 is a SUMOylation site. SUMOylation at this site is responsible for the transcription repression activity of K-bZIP [27,51]. This is consistent with our current finding that viral gene expression is elevated with loss of SUMO-2/3 modification (Fig 4B). However, there may be multiple potential SUMO-2/3 targets that are responsible for global SUMO-2/3 enrichment on the viral genome during reactivation. The factors responsible for SUMO-2/3 enrichment on the host genome can be more complex. Several potential SUMO-2/3 targets we identified in our recent study provide clues for identifying proteins that are responsible for global SUMO-2/3 enrichment on the host genome [10]. Moreover, we cannot exclude the possibility that those same factors may also bind to the viral genome and contribute to the SUMO-2/3 enrichment on the KSHV genome. This is a very interesting topic for further study as it can explore how SUMO modification functions in the regulation of epigenetic status.

**Fig 10:**
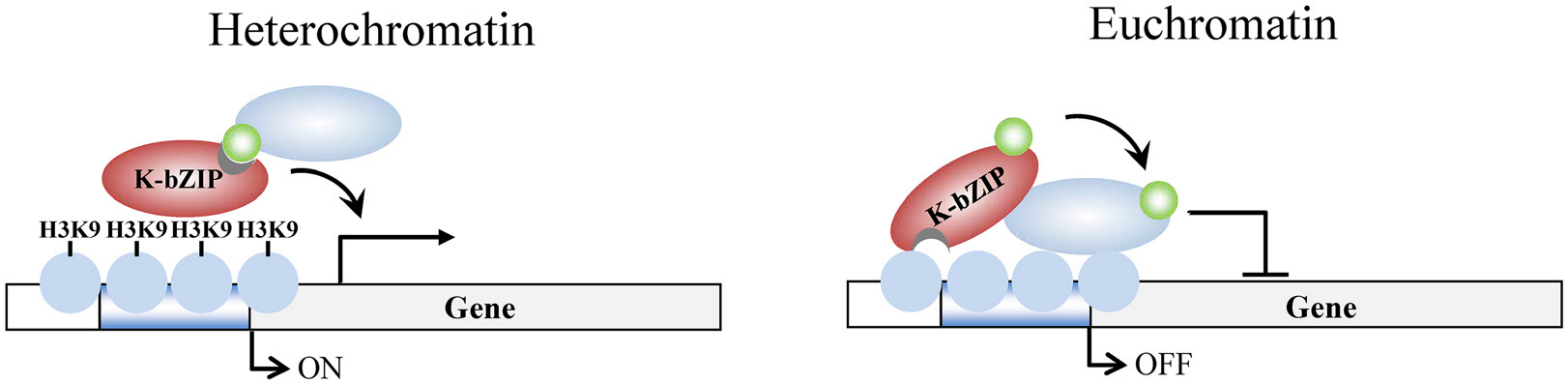
Model of K-bZIP regulation of viral gene expression in heterochromatin and euchromatin regions of the KSHV genome during reactivation. In heterochromatin region, K-bZIP binds directly to H3K9me3 and modulates the viral gene activation by recruiting SUMOylated transcription regulators. In euchromatin region, K-bZIP binds directly or indirectly (through interacting with other transcription factors) to DNA and mediates SUMOylation-dependent transcription repression. Green circles, SUMO-2/3; blue ovals, unknown SUMOylated target; brown ovals, K-bZIP; blue circles, histones; H3K9, histone H3K9me3 mark.

Surprisingly, during reactivation in K-bZIP-L75A mutated rKSHV, the viral genes in the high H3K9me3 mark region where there is little SUMO-2/3 enrichment, showed defective transcription activity. This implies that K-bZIP may participate in transactivation of viral genes located in high H3K9me3 regions in a SUMO E3 ligase-independent manner. Although K-bZIP is widely accepted as a transcriptional repressor, K-bZIP has also been found to activate gene transcription through direct DNA binding [52]. Direct K-bZIP binding results in a low level of gene transcription. Consistent with our current finding showing relatively low expression level of genes in high H3K9me3 region of KSHV genome (Fig 3), K-bZIP may directly bind to some of the viral promoters located in this heterochromatin region and mediate a low level of gene transactivation (Fig 10). This hypothesis is further supported by our recent finding showing a direct binding of K-bZIP to H3K9me3 [17]. The difference in epigenetic context may provide an explanation for the opposing functions of K-bZIP in transcription regulation. Consistent with this notion, a previous report of cytomegalovirus showed that the SIM of IE2 is required for recruiting a SUMOylated transcription initiation factor and this recruitment is essential for the transactivation function of IE2 [53]. Our data here showed that this SIM-dependent recruitment phenomenon may also be true for KSHV K-bZIP. The underlying mechanism for SUMOylation-independent SIM-mediated transactivation in K-bZIP is interesting, however, it still requires for further study.

In addition to transcription regulation, the role of K-bZIP in KSHV replication is also controversial. Several reports have showed that K-bZIP is able to regulate KSHV replication through interacting and regulating Ori-Lyt-binding proteins, such as LANA [54] or HDAC [34]. Though it has also been reported that K-bZIP can directly bind to KSHV Ori-Lyt [55,56], the same group showed that K-bZIP is not absolutely required for Ori-Lyt-mediated KSHV DNA replication [36]. In contrast, other studies showed that K-bZIP is essential for Ori-Lyt-mediated replication and virion production [35,57]. However, one group also reported that over-expression of K-Rta can overcome the absence of K-bZIP [58]. It has been long known that K-bZIP and K-Rta play an antagonist role against each other. K-Rta, the major transactivator of KSHV, was recently identified as a SUMO-Targeted Ubiquitin Ligase (STUbL). The STUbL activity of K-Rta is a prerequisite for its transactivation activity and for reactivation of KSHV [59]. Consistent with the notion that K-bZIP may oppose the activating function of K-Rta, we found that the E3 ligase activity is essential for K-bZIP to repress activation of KSHV lytic genes and virus reactivation. This finding uncovers a novel mechanism of antagonism between K-Rta and K-bZIP in regulating KSHV life cycle.

Although SUMOylation of individual transcription factors is responsible for transcription repression, global SUMO modification of chromatin was found to be essential for either activation of genes [11] or shut-off of active genes after induction [5,10]. The multiple isoforms of SUMO proteins may contribute to this discrepancy. However, a recent study on global SUMO modifications of the human genome consistently showed similar epigenetic alterations between SUMO paralogs under physiological stimuli tested [12]. Using KSHV as a model, a SUMO-2/3 specific function in epigenetic regulation of transcription has been revealed. To our knowledge, this is the first report showing that a virus targets SUMO-2/3 specifically for epigenetic modification of its genome and repression of its lytic gene expression. Our results suggest that viruses have evolved a unique way to hijack the SUMO machinery in a paralog-specific manner to alleviate reactivation that may benefit their own survival.

## Supporting Information

S1 Fig Expression of K-bZIP after TPA and Dox induced KSHV reactivation. Immunoblotting was performed using TCLs prepared from TREx-F3H3-K-Rta BCBL-1 cells; untreated, or treated with 20 ng/ml TPA and 0.2 μg/ml Dox for the time period shown. Blots were probed with anti-Flag and anti-K-bZIP antibodies. Anti-GAPDH antibody was used for loading control.
(TIFF)

S2 Fig SUMO-2/3 modification on KSHV promoters representing high SUMO-2/3 enrichment or H3K9me3-rich regions in viral lytic genome. (A) The expression of SUMO-1 and SUMO-2/3 before and after Dox induction for viral reactivation was analyzed using anti-SUMO-1 and anti-SUMO-2/3 antibody, respectively. Ratio for each blot is the signal/α-tubulin observed at 12 hours Dox treatment setting 0 hour Dox as 1.0. Immunoblotting using anti-K-Rta antibody to confirm the induction of K-Rta in TREx-F3H3-K-Rta BCBL-1 cells after 0.2 μg/ml Dox treatment for 12 hours. Anti-α-tubulin antibody was used for loading control. (B) ChIP assay for SUMO paralogs was performed using chromatin prepared from non-induced (0 hour) and 0.2 μg/ml Dox-treated (12 hours) TREx-F3H3-K-Rta BCBL-1 cells using anti-SUMO-1 or SUMO-2/3 antibody. SUMO-1 and SUMO-2/3 binding to promoters in SUMO-2/3 enrichment and H3K9me3-rich regions were analyzed by real-time qPCR. ChIP enrichment was computed by comparing values obtained from Dox-treated samples to their non-induced controls. Rabbit IgG was used as negative antibody control and IgG enrichment is very low or not visible in both ChIP assays.
(TIFF)

S3 Fig Deposition of SUMO-1 and SUMO-2 modification on selected KSHV promoters representing high SUMO-2/3 enrichment or H3K9me3-rich regions during reactivation. (A) TCLs from non-induced (0 hour) and 0.2 μg/ml Dox-treated (12 hours) TREx-MH-K-Rta BCBL-1 and TREx-MH-K-Rta-Flag-SUMO-1 and -Flag-SUMO-2 BCBL-1 cells were analyzed by immunoblotting using anti-Flag and anti-K-Rta antibodies. Anti-α-tubulin antibody was used for loading control. Ratio for each construct is the Flag/α-tubulin signal observed for Dox treatment at 12 hour using Dox at 0 hour set as 1.0. (B) ChIP was performed using chromatin prepared from cells treated as described in (A) using anti-Flag antibody. Rabbit IgG was used as negative antibody control and enrichment is not visible in most cases. SUMO-1 and SUMO-2 binding to SUMO-2/3 enrichment and H3K9me3-rich regions were analyzed by real-time qPCR using primer pairs as described in Fig 2.
(TIFF)

S4 Fig Knockdown SUMO-2/3 does not induce the expression of viral lytic genes located in the high SUMO-2/3 enrichment region in latent KSHV infected BCBL-1 cells. (A) pLenti4-shSUMO-2 and -3 were introduced into TREx-BCBL-1 cells by lentiviral transduction. 0.2 μg/ml of Dox was added to cells 48 hours after transduction to induce SUMO-2/3 knockdown. TCLs were collected 24 hours after Dox treatment and analyzed by immunoblotting using anti-SUMO-2/3 antibody. Anti-α-tubulin antibody was used for loading control. (B and C) Total RNA isolated from cells treated as described in (A) was reverse transcribed using oligo-d(T)_18_ primer. The expression level of SUMO-2, SUMO-3 (B), and four viral genes representing SUMO-2/3 enrichment region as described in Fig 1 (C) were quantified by real-time qPCR. All reactions were run in triplicate and normalized against GAPDH. The fold was computed by using non-induced controls as 1.0. **; P<0.005. NS; non-significant.
(TIFF)

S5 Fig SUMO-2/3 knockdown in SUMO-2/3 inducible knockdown BCBL-1 cells. TCLs isolated from non-induced (0 hour) and 0.2 μg/ml Dox-induced (for 12, 24 and 48 hours) TREx-F3H3-K-Rta BCBL-1 and TREx-F3H3-K-Rta-shSUMO-2/3 BCBL-1 cells were subjected to immunoblotting analysis using anti-SUMO-2/3 antibody. Induction of K-Rta and expression of K-bZIP was confirmed by using anti-K-Rta and anti-K-bZIP antibodies. Anti-GAPDH antibody was used for loading control. Ratio for each cell line is the SUMO-2/3/GAPDH signal observed for Dox treatment at 0 (for shSUMO-2/3), 12, 24, and 48 hour using TREx-F3H3-K-Rta BCBL-1 cells at 0 hour set as 1.0.
(TIFF)

S6 Fig Knockdown of SUMO-2/3 increases transactivation of KSHV lytic genes located in the SUMO-2/3 enrichment and high H3K9me3 region but not in latent genes located in SUMO-2/3 enrichment and high SUMO-1 region. (A) The expression level of three viral lytic genes representing the SUMO-2/3 enrichment and high H3K9me3 region were quantified by real-time qPCR using cDNA from Fig 4A. All reactions were run in triplicate and normalized against GAPDH. The fold change was computed by comparing induced values to their non-induced controls. **; P<0.005. NS; non-significant. (B) The expression level of three viral latent genes representing the SUMO-2/3 enrichment and high SUMO-1 region were quantified as described in (A).
(TIFF)

S7 Fig SUMO-2/3 knockdown does not change histone marks on the KSHV genome during viral reactivation. (A) TREx-F3H3-K-Rta BCBL-1 and TREx-F3H3-K-Rta-shSUMO-2/3 BCBL-1 cells were treated as described in Fig 4A. TCLs were collected and analyzed by immunoblotting using anti-SUMO-2/3, anti-K-Rta and anti-K-bZIP antibodies. Anti-GAPDH antibody was used for loading control. (B) ChIP assays were performed using chromatin prepared from cells treated as described in (A) using anti-H3K9me3, anti-H3K27me3, anti-H3K4me3, anti-H3K36me3, and anti-H3Ac antibodies. Rabbit IgG was used as negative antibody control. Histone marks on the K-bZIP promoter region that were highly enriched by SUMO-2/3 were analyzed by real-time qPCR.
(TIFF)

S8 Fig Generation of iSLK-Puro-BAC16 K-bZIP-WT rev and -L75A cell lines. (A) Western blot analysis of LANA expression in iSLK-Puro-BAC16 K-bZIP-WT rev and -L75A cells. GAPDH was probed as control. Ratio for each cell line is the LANA/GAPDH signal observed for Dox treatment at 24 and 48 hour using Dox at 0 hour set as 1.0. (B) Genomic DNA from iSLK-Puro-BAC16 K-bZIP-WT rev and -L75A cells was prepared using phenol/chloroform extraction and KSHV genome copy was determined by real-time qPCR using orf19 and orf20 specific primer pairs. A primer pair specific for the promoter region of cellular gene RTP4 was used as control. The fold was computed by comparing K-bZIP-L75A copy number values to K-bZIP-WT rev.
(TIFF)

S9 Fig K-bZIP binding on selected KSHV promoters in iSLK-Puro-BAC16 K-bZIP-WT rev and -L75A cell lines. ChIP for K-bZIP was performed using chromatin prepared from non-induced (0 hour) and 1 μg/ml Dox-treated (24 hours) iSLK-Puro-BAC16 K-bZIP-WT rev and -L75A cells using anti-K-bZIP antibody or rabbit IgG. K-bZIP binding to promoters in SUMO-2/3 enrichment and H3K9me3-rich regions was analyzed by real-time qPCR. Enrichment using rabbit IgG negative antibody control is not visible in some plots.
(TIFF)

S10 Fig K-Rta-expressing cells show more lytic protein expression in iSLK-Puro-BAC16 K-bZIP-L75A cells compared to iSLK-Puro-BAC16 K-bZIP-WT rev during KSHV reactivation. (A) iSLK-Puro-BAC16 K-bZIP-WT rev and -L75A cells were treated with 1 μg/ml Dox for 48 hours. Non-induced (-Dox) and Dox-treated (+Dox) cells were fixed by 4% paraformaldehyde and stained using anti-K-Rta and anti-K-bZIP antibodies. Representative immunofluorescence assay (IFA) stained images showing K-bZIP positive cells in K-Rta expressing cells. (B) The K-bZIP positive cells in K-Rta expressing cells were quantified from >20 microscopic fields. Number of K-bZIP positive cells (pink) among K-Rta expressing cells (red) was calculated as % of K-Rta positive cells. *; P<0.05
(TIFF)

S11 Fig The SUMO E3 ligase activity of K-bZIP is essential for diminution of virus production during reactivation. (A) The expression of K-Rta and K-bZIP in iSLK-Puro harboring parental BAC16 clone, iSLK-Puro-BAC16 K-bZIP-WT, and iSLK-Puro-BAC16 K-bZIP-WT rev and -L75A cells before and after Dox induction for 24 hours was analyzed by immunoblotting. GAPDH was probed as control. (B) Supernatants harvested from iSLK-Puro-BAC16 K-bZIP-WT, -WT rev and -L75A cells treated with or without Dox for 72 hours were filtered and used to infect 293T cells. GFP positive cells were analyzed by fluorescence microscopy (FITC, 10X magnification) 48 hours after infection. (C) The GFP positive cells were quantified using the average from >20 microscopic fields. ***; P<0.001. NS; non-significant.
(TIFF)

S12 Fig SUMO E3 ligase activity of K-bZIP does not essential for H3K9me3 mark on KSHV genome during lytic reactivation. (A) TCLs were collected from iSLK-Puro-BAC16 K-bZIP-WT rev and -L75A cells treated as described in Fig 9 and analyzed using anti-K-Rta and anti-K-bZIP antibodies. Anti-GAPDH antibody was used for loading control. (B) ChIP was performed using chromatin prepared as described in (A) using anti-H3K9me3 antibody. Rabbit IgG was used as negative antibody control.
(TIFF)

S1 Table Primer sequence for ChIP-qPCR. (DOCX)

S2 Table Primer sequence for RT-qPCR. (DOCX)
