# Supplementary figures and images for: K-bZIP Mediated SUMO-2/3 Specific Modification on the KSHV Genome Negatively Regulates Lytic Gene Expression and Viral Reactivation
Source: PLoS Pathog. 2015 Jul 21;11(7):e1005051. doi: 10.1371/journal.ppat.1005051 (PMC4510548; doi:10.1371/journal.ppat.1005051)

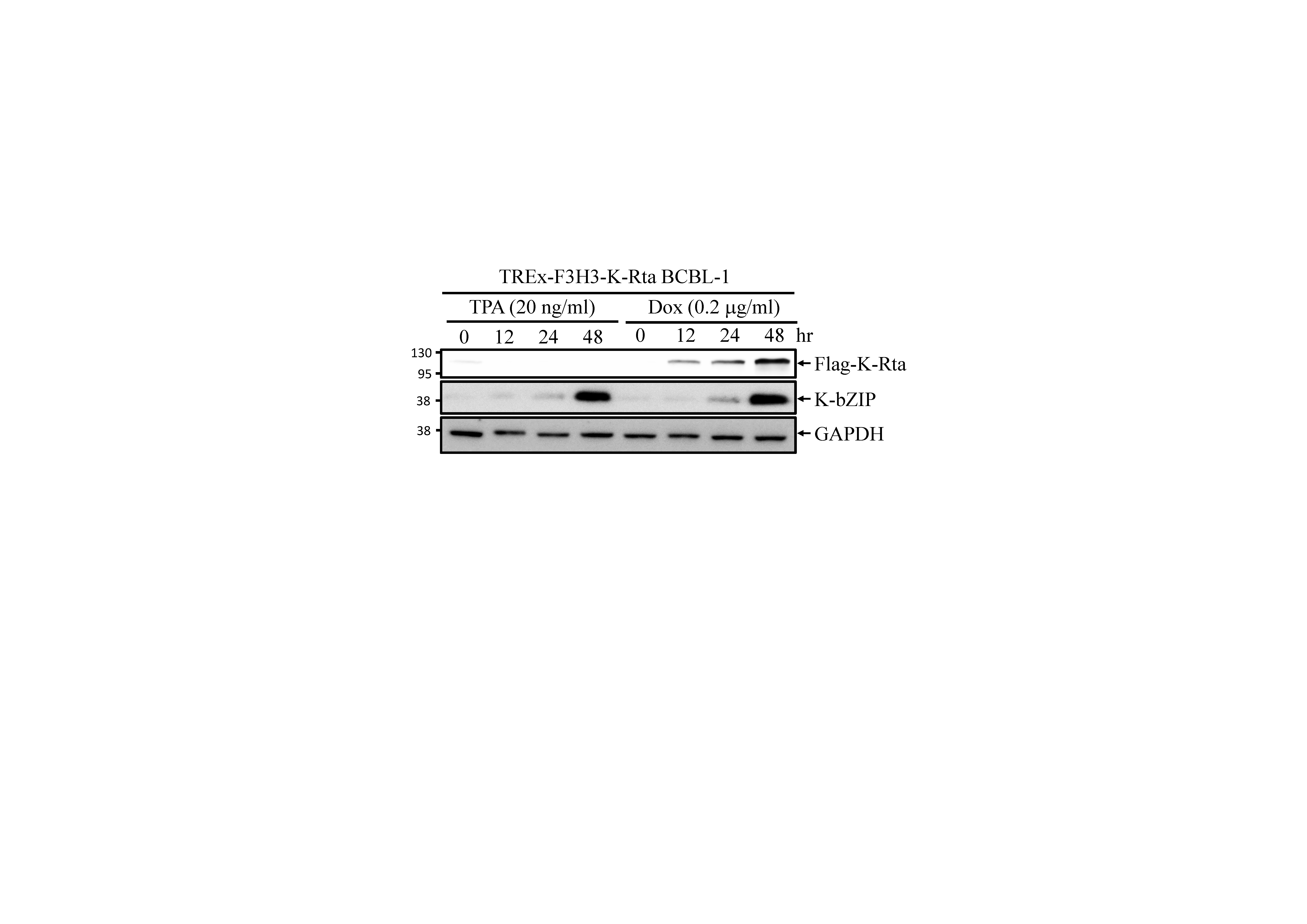

Supplement: S1 Fig — Immunoblotting was performed using TCLs prepared from TREx-F3H3-K-Rta BCBL-1 cells; untreated, or treated with 20 ng/ml TPA and 0.2 μg/ml Dox for the time period shown. Blots were probed with anti-Flag and anti-K-bZIP antibodies. Anti-GAPDH antibody was used for loading control. (TIFF) [file ppat.1005051.s001.tiff]

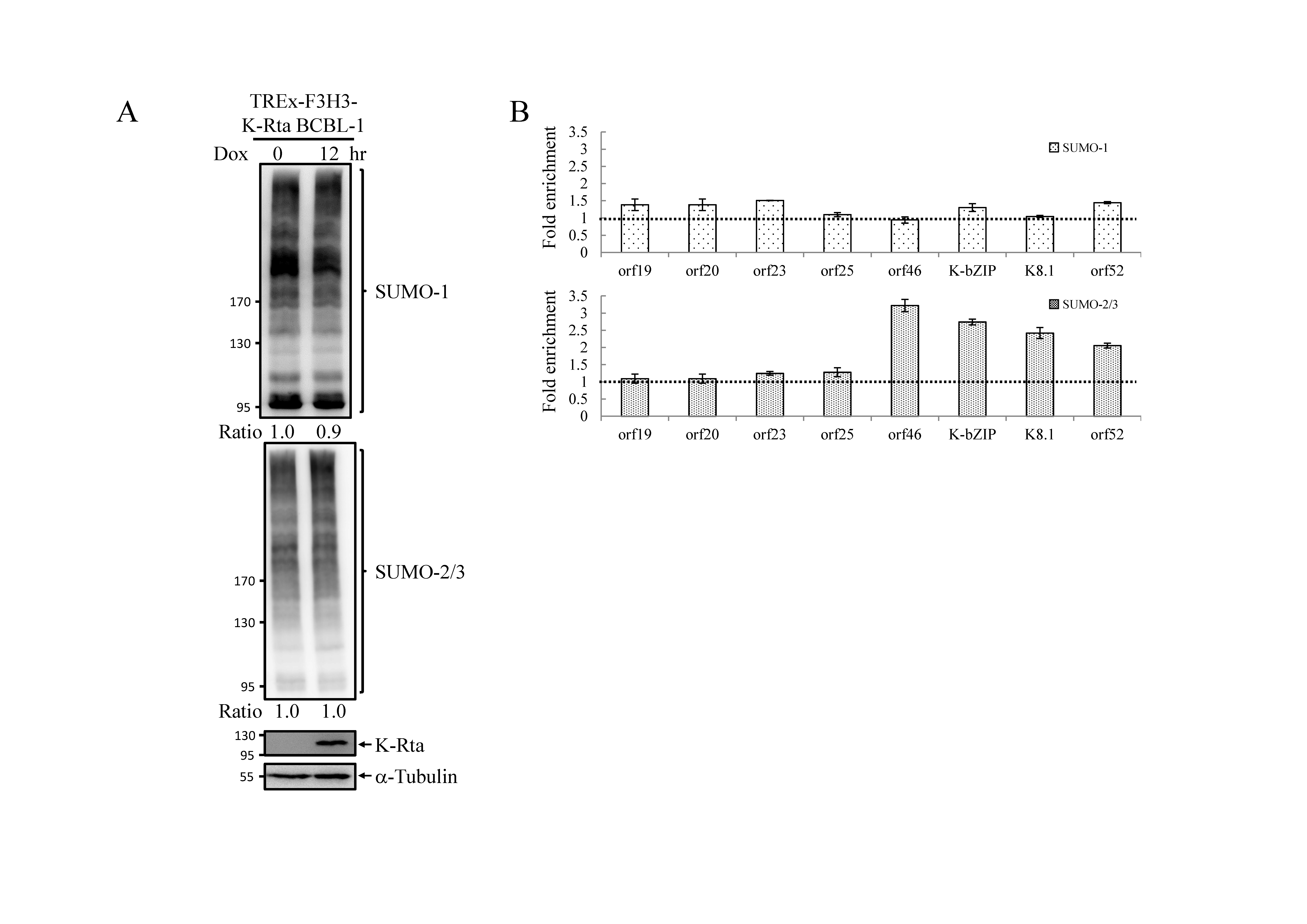

Supplement: S2 Fig — (A) The expression of SUMO-1 and SUMO-2/3 before and after Dox induction for viral reactivation was analyzed using anti-SUMO-1 and anti-SUMO-2/3 antibody, respectively. Ratio for each blot is the signal/α-tubulin observed at 12 hours Dox treatment setting 0 hour Dox as 1.0. Immunoblotting using anti-K-Rta antibody to confirm the induction of K-Rta in TREx-F3H3-K-Rta BCBL-1 cells after 0.2 μg/ml Dox treatment for 12 hours. Anti-α-tubulin antibody was used for loading control. (B) ChIP assay for SUMO paralogs was performed using chromatin prepared from non-induced (0 hour) and 0.2 μg/ml Dox-treated (12 hours) TREx-F3H3-K-Rta BCBL-1 cells using anti-SUMO-1 or SUMO-2/3 antibody. SUMO-1 and SUMO-2/3 binding to promoters in SUMO-2/3 enrichment and H3K9me3-rich regions were analyzed by real-time qPCR. ChIP enrichment was computed by comparing values obtained from Dox-treated samples to their non-induced controls. Rabbit IgG was used as negative antibody control and IgG enrichment is very low or not visible in both ChIP assays. (TIFF) [file ppat.1005051.s002.tiff]

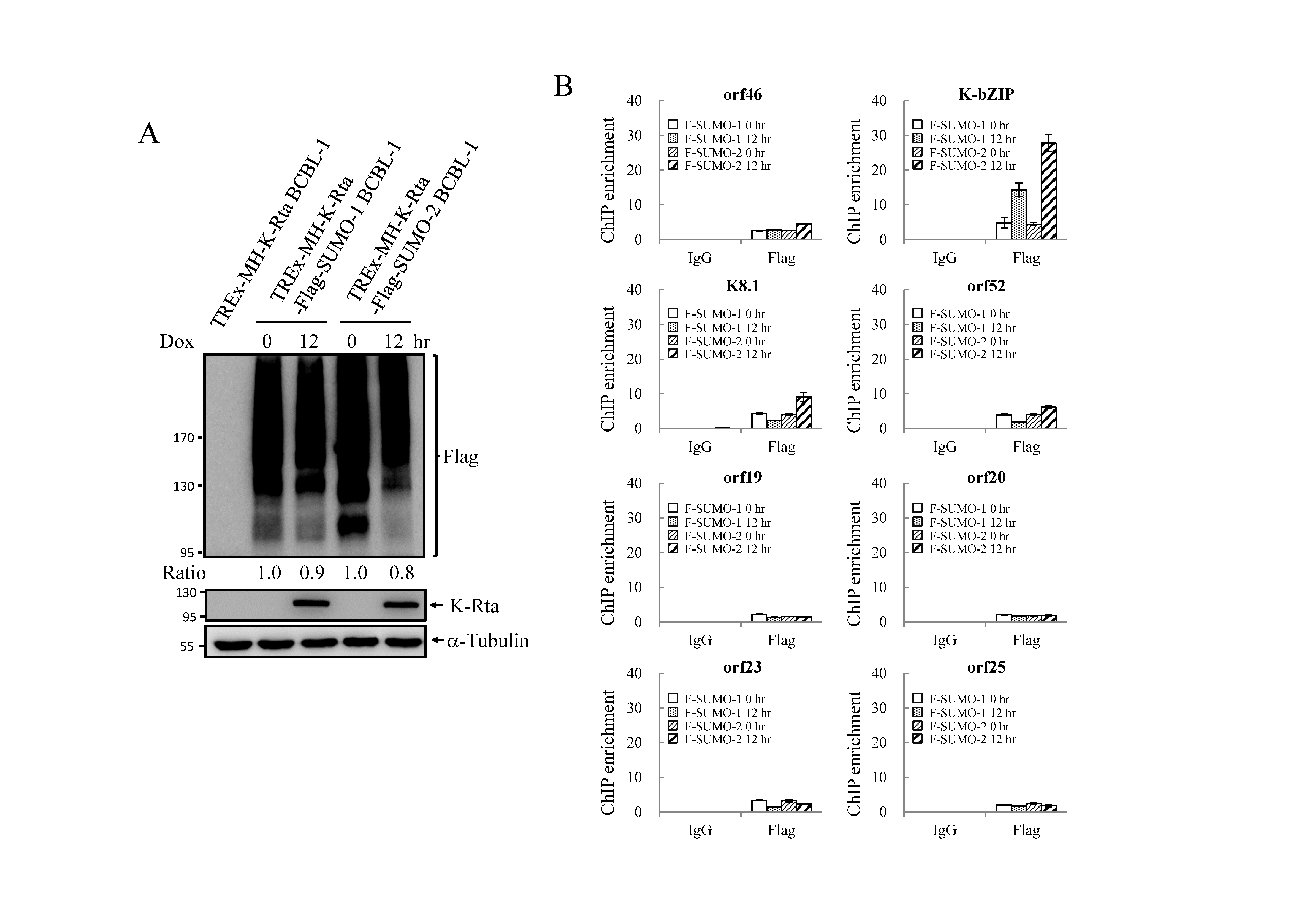

Supplement: S3 Fig — (A) TCLs from non-induced (0 hour) and 0.2 μg/ml Dox-treated (12 hours) TREx-MH-K-Rta BCBL-1 and TREx-MH-K-Rta-Flag-SUMO-1 and -Flag-SUMO-2 BCBL-1 cells were analyzed by immunoblotting using anti-Flag and anti-K-Rta antibodies. Anti-α-tubulin antibody was used for loading control. Ratio for each construct is the Flag/α-tubulin signal observed for Dox treatment at 12 hour using Dox at 0 hour set as 1.0. (B) ChIP was performed using chromatin prepared from cells treated as described in (A) using anti-Flag antibody. Rabbit IgG was used as negative antibody control and enrichment is not visible in most cases. SUMO-1 and SUMO-2 binding to SUMO-2/3 enrichment and H3K9me3-rich regions were analyzed by real-time qPCR using primer pairs as described in Fig 2. (TIFF) [file ppat.1005051.s003.tiff]

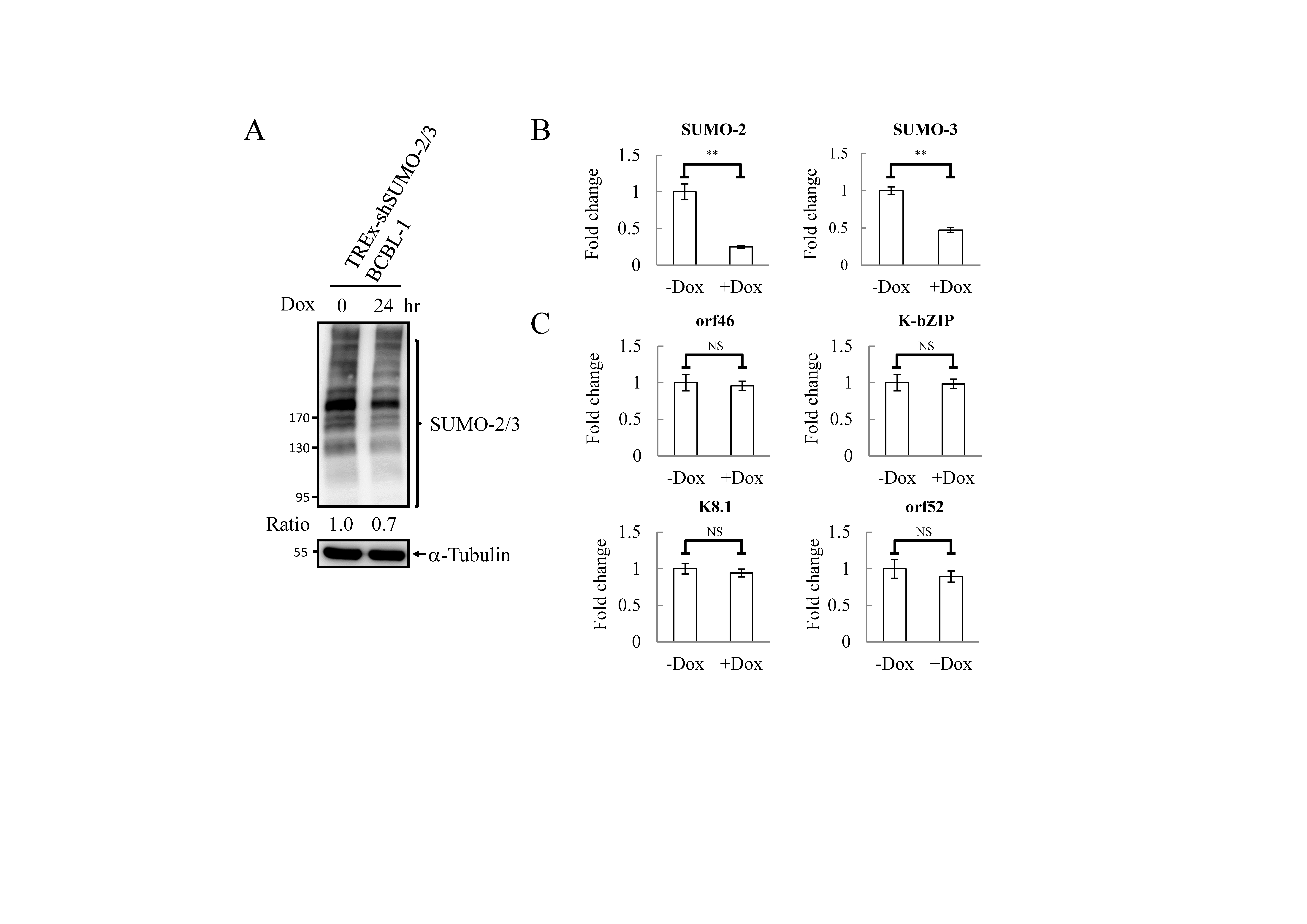

Supplement: S4 Fig — (A) pLenti4-shSUMO-2 and -3 were introduced into TREx-BCBL-1 cells by lentiviral transduction. 0.2 μg/ml of Dox was added to cells 48 hours after transduction to induce SUMO-2/3 knockdown. TCLs were collected 24 hours after Dox treatment and analyzed by immunoblotting using anti-SUMO-2/3 antibody. Anti-α-tubulin antibody was used for loading control. (B and C) Total RNA isolated from cells treated as described in (A) was reverse transcribed using oligo-d(T)18 primer. The expression level of SUMO-2, SUMO-3 (B), and four viral genes representing SUMO-2/3 enrichment region as described in Fig 1 (C) were quantified by real-time qPCR. All reactions were run in triplicate and normalized against GAPDH. The fold was computed by using non-induced controls as 1.0. **; P<0.005. NS; non-significant. (TIFF) [file ppat.1005051.s004.tiff]

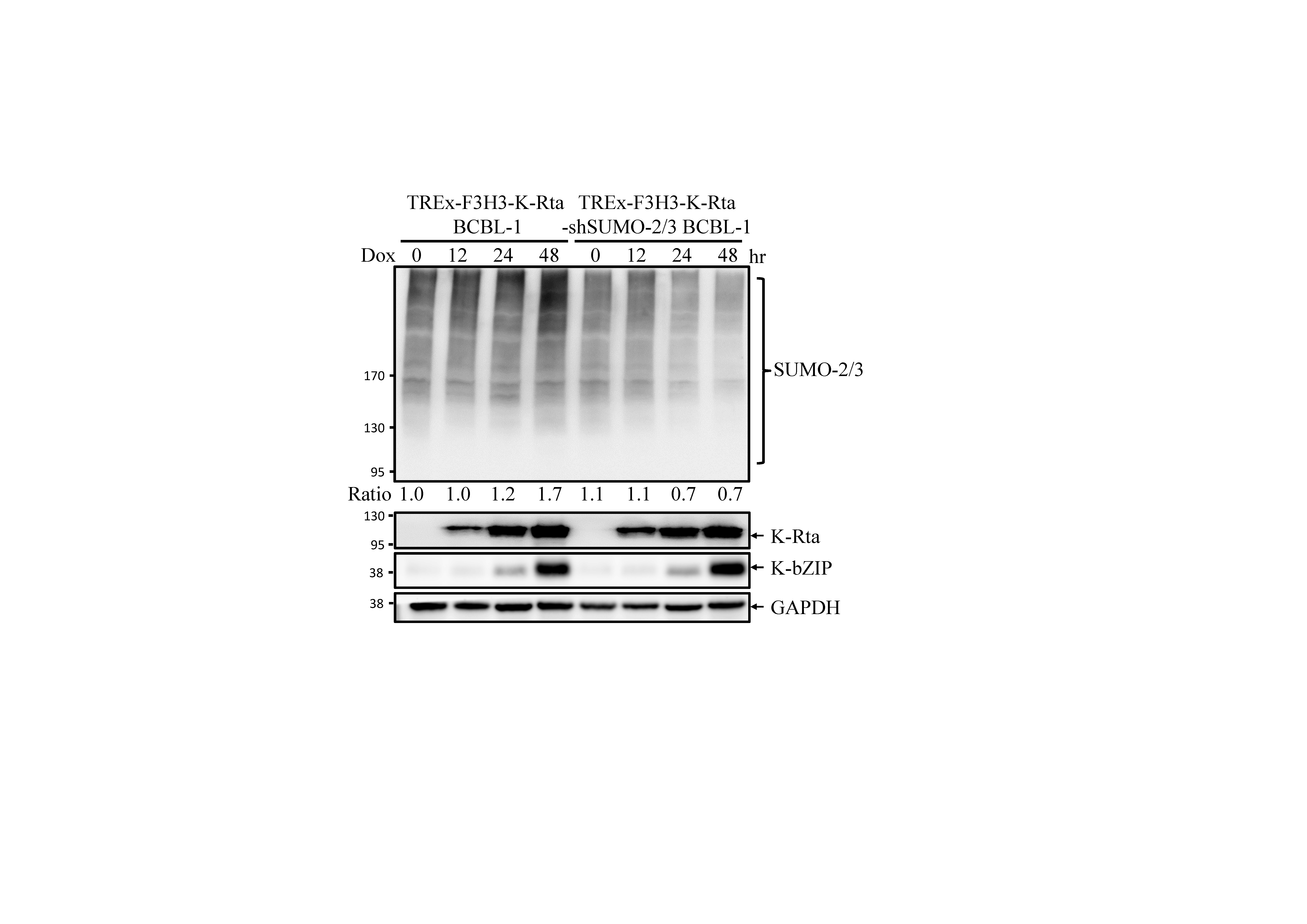

Supplement: S5 Fig — TCLs isolated from non-induced (0 hour) and 0.2 μg/ml Dox-induced (for 12, 24 and 48 hours) TREx-F3H3-K-Rta BCBL-1 and TREx-F3H3-K-Rta-shSUMO-2/3 BCBL-1 cells were subjected to immunoblotting analysis using anti-SUMO-2/3 antibody. Induction of K-Rta and expression of K-bZIP was confirmed by using anti-K-Rta and anti-K-bZIP antibodies. Anti-GAPDH antibody was used for loading control. Ratio for each cell line is the SUMO-2/3/GAPDH signal observed for Dox treatment at 0 (for shSUMO-2/3), 12, 24, and 48 hour using TREx-F3H3-K-Rta BCBL-1 cells at 0 hour set as 1.0. (TIFF) [file ppat.1005051.s005.tiff]

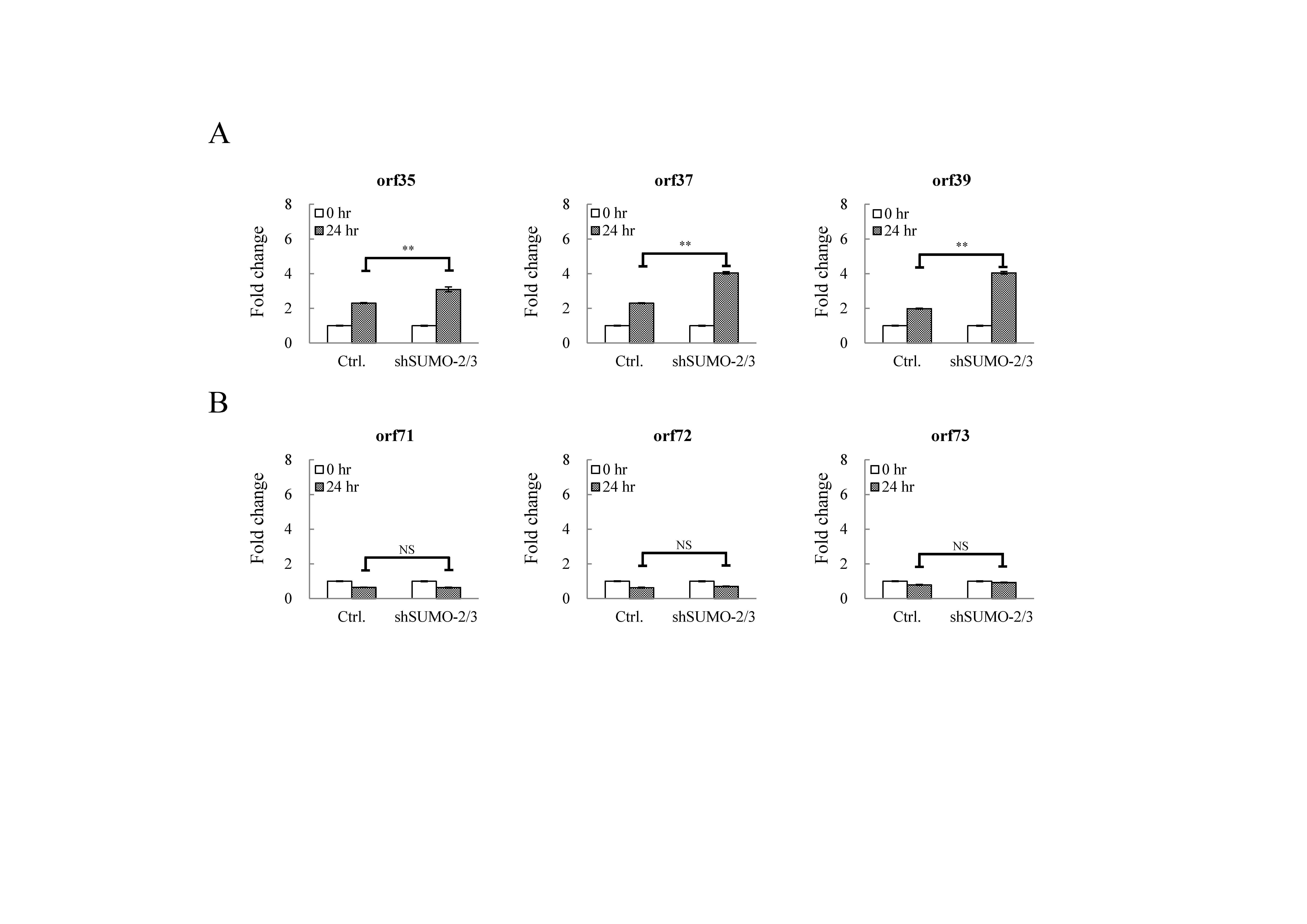

Supplement: S6 Fig — (A) The expression level of three viral lytic genes representing the SUMO-2/3 enrichment and high H3K9me3 region were quantified by real-time qPCR using cDNA from Fig 4A. All reactions were run in triplicate and normalized against GAPDH. The fold change was computed by comparing induced values to their non-induced controls. **; P<0.005. NS; non-significant. (B) The expression level of three viral latent genes representing the SUMO-2/3 enrichment and high SUMO-1 region were quantified as described in (A). (TIFF) [file ppat.1005051.s006.tiff]

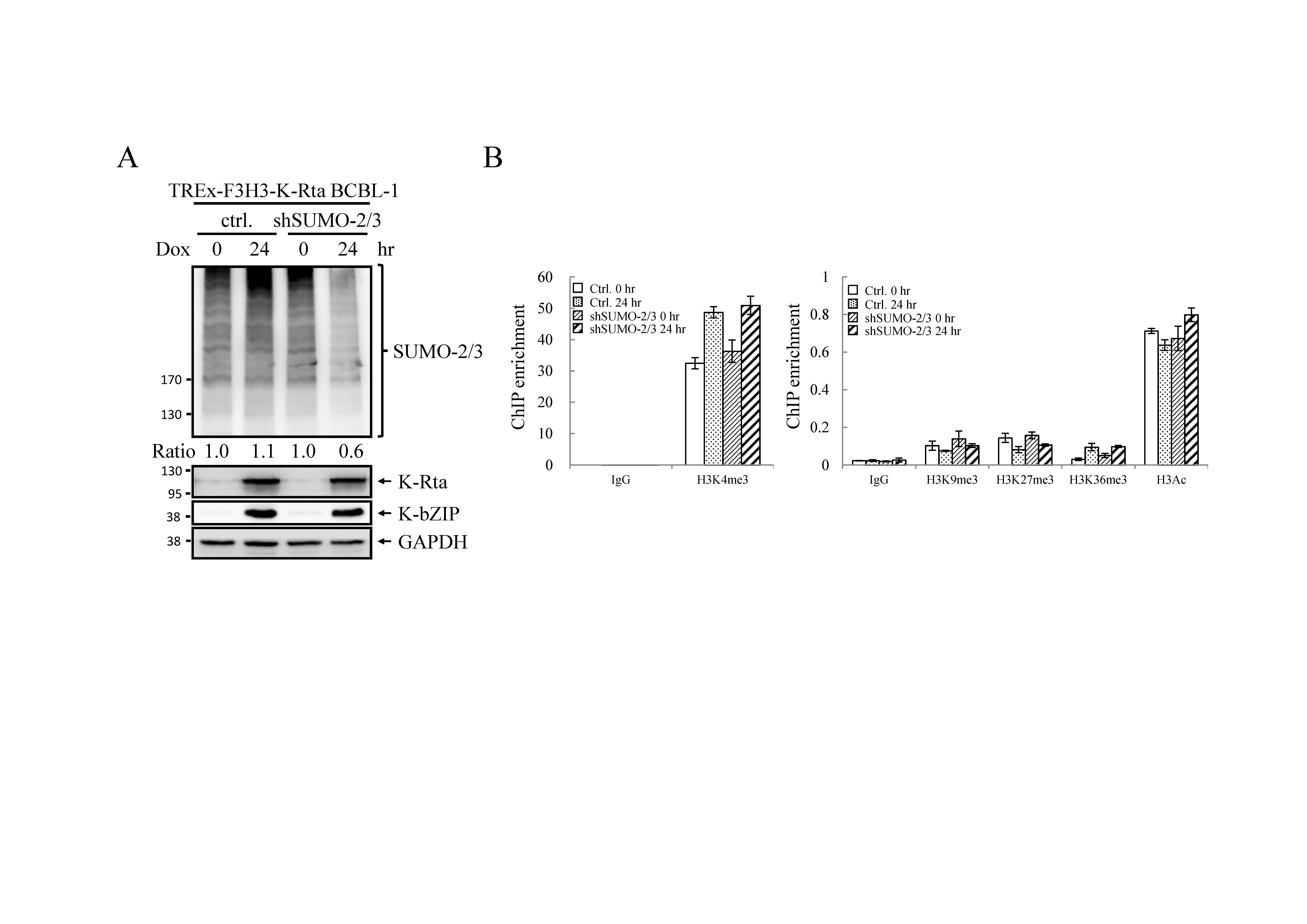

Supplement: S7 Fig — (A) TREx-F3H3-K-Rta BCBL-1 and TREx-F3H3-K-Rta-shSUMO-2/3 BCBL-1 cells were treated as described in Fig 4A. TCLs were collected and analyzed by immunoblotting using anti-SUMO-2/3, anti-K-Rta and anti-K-bZIP antibodies. Anti-GAPDH antibody was used for loading control. (B) ChIP assays were performed using chromatin prepared from cells treated as described in (A) using anti-H3K9me3, anti-H3K27me3, anti-H3K4me3, anti-H3K36me3, and anti-H3Ac antibodies. Rabbit IgG was used as negative antibody control. Histone marks on the K-bZIP promoter region that were highly enriched by SUMO-2/3 were analyzed by real-time qPCR. (TIFF) [file ppat.1005051.s007.tiff]

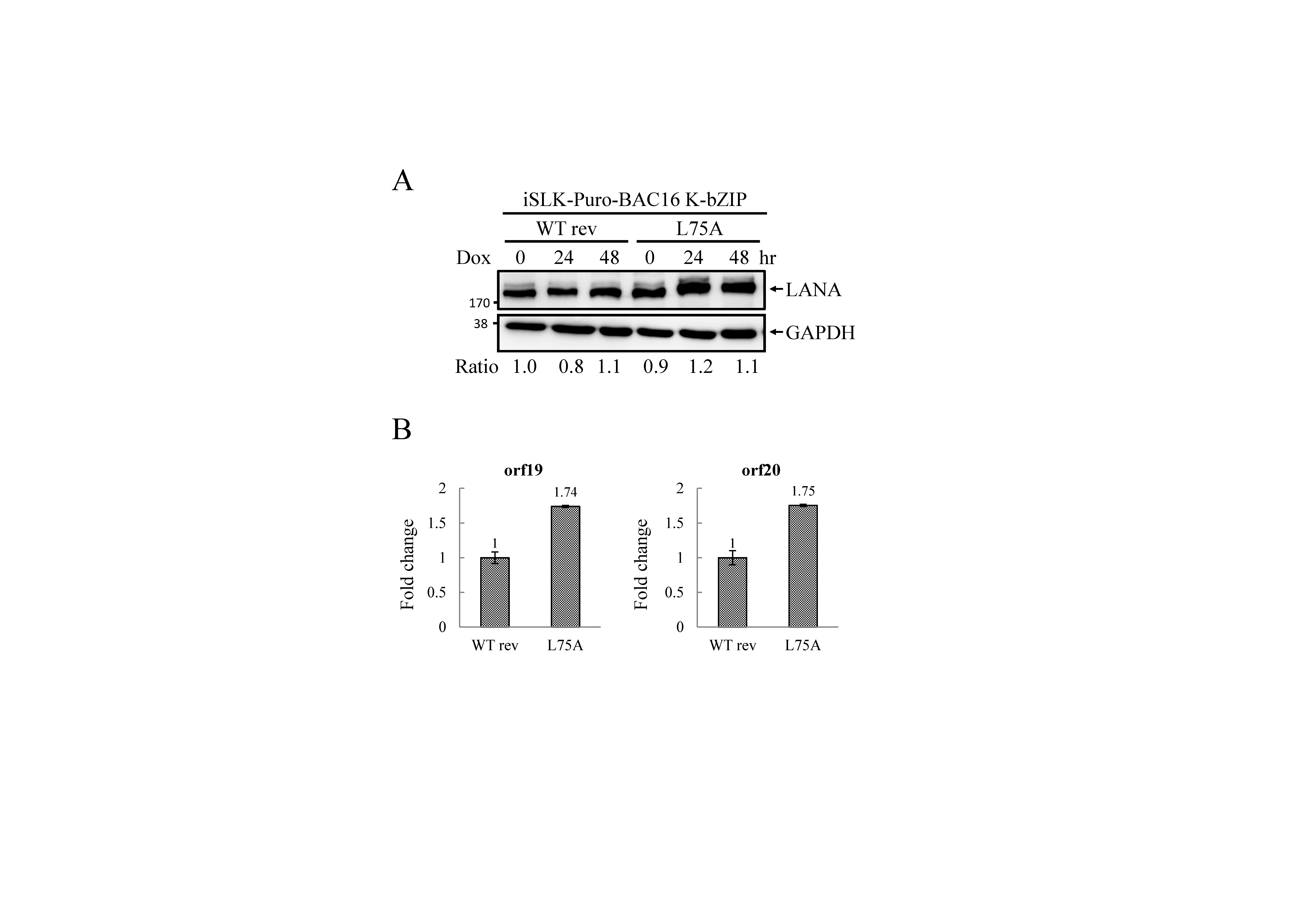

Supplement: S8 Fig — (A) Western blot analysis of LANA expression in iSLK-Puro-BAC16 K-bZIP-WT rev and -L75A cells. GAPDH was probed as control. Ratio for each cell line is the LANA/GAPDH signal observed for Dox treatment at 24 and 48 hour using Dox at 0 hour set as 1.0. (B) Genomic DNA from iSLK-Puro-BAC16 K-bZIP-WT rev and -L75A cells was prepared using phenol/chloroform extraction and KSHV genome copy was determined by real-time qPCR using orf19 and orf20 specific primer pairs. A primer pair specific for the promoter region of cellular gene RTP4 was used as control. The fold was computed by comparing K-bZIP-L75A copy number values to K-bZIP-WT rev. (TIFF) [file ppat.1005051.s008.tiff]

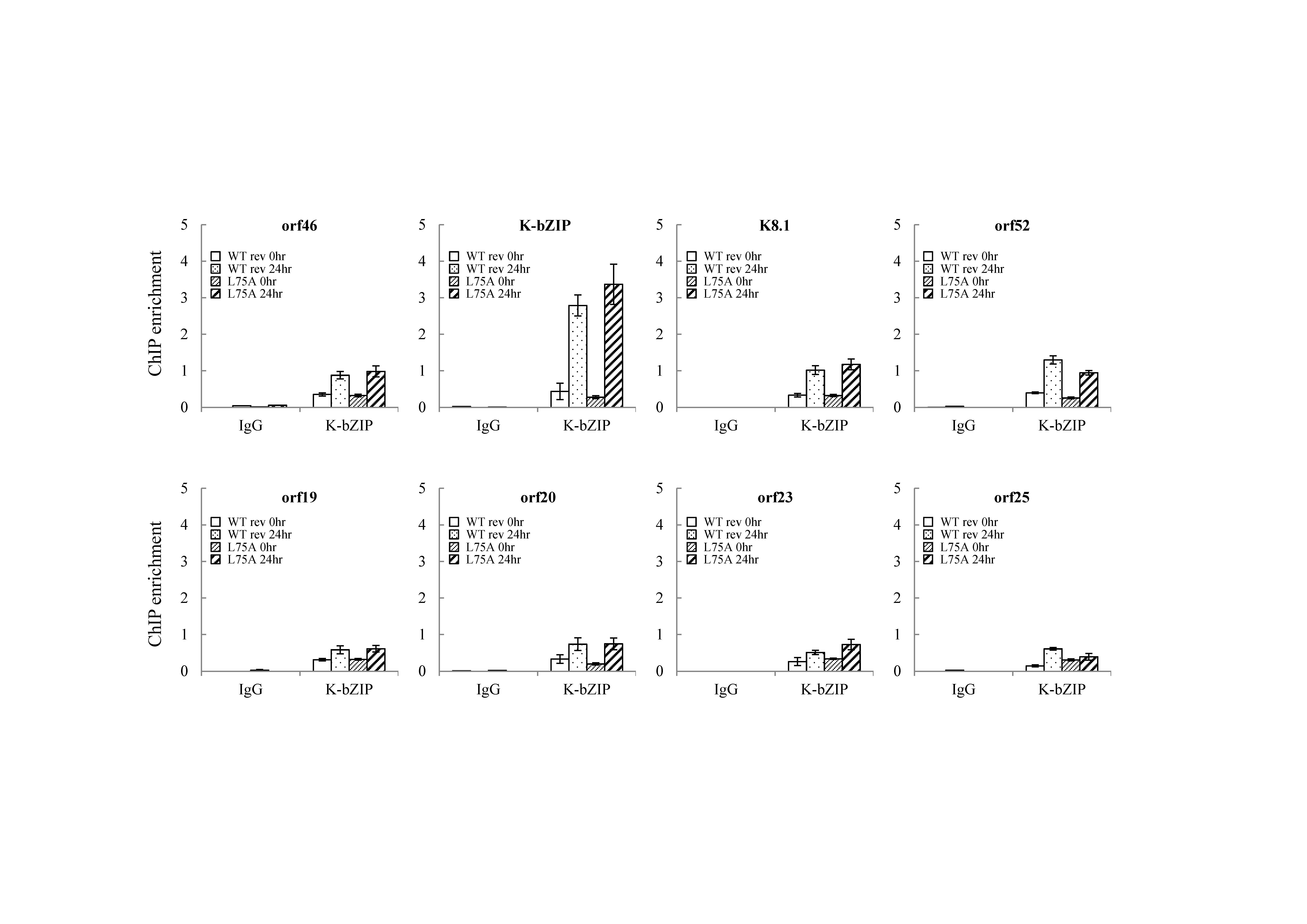

Supplement: S9 Fig — ChIP for K-bZIP was performed using chromatin prepared from non-induced (0 hour) and 1 μg/ml Dox-treated (24 hours) iSLK-Puro-BAC16 K-bZIP-WT rev and -L75A cells using anti-K-bZIP antibody or rabbit IgG. K-bZIP binding to promoters in SUMO-2/3 enrichment and H3K9me3-rich regions was analyzed by real-time qPCR. Enrichment using rabbit IgG negative antibody control is not visible in some plots. (TIFF) [file ppat.1005051.s009.tiff]

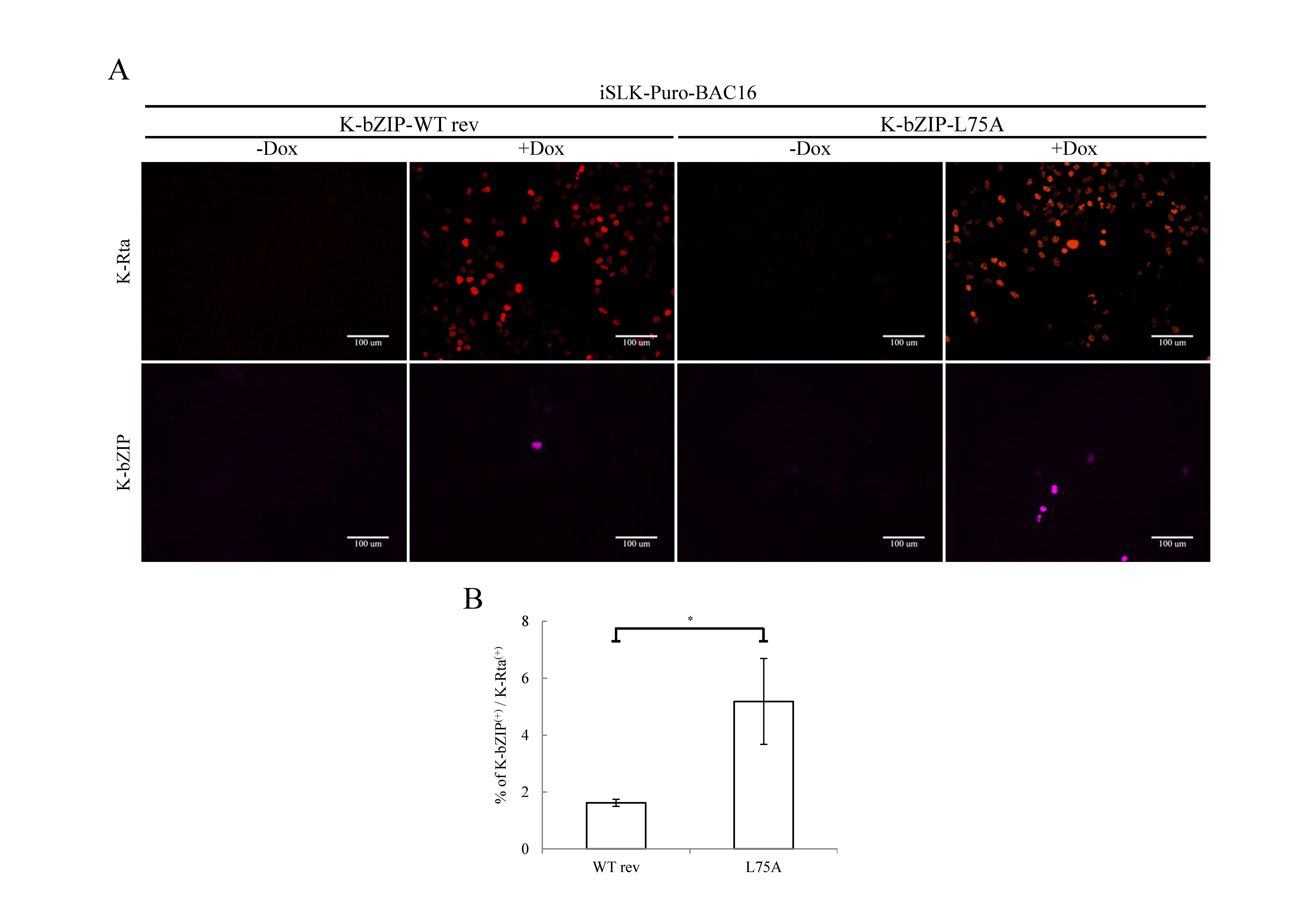

Supplement: S10 Fig — (A) iSLK-Puro-BAC16 K-bZIP-WT rev and -L75A cells were treated with 1 μg/ml Dox for 48 hours. Non-induced (-Dox) and Dox-treated (+Dox) cells were fixed by 4% paraformaldehyde and stained using anti-K-Rta and anti-K-bZIP antibodies. Representative immunofluorescence assay (IFA) stained images showing K-bZIP positive cells in K-Rta expressing cells. (B) The K-bZIP positive cells in K-Rta expressing cells were quantified from >20 microscopic fields. Number of K-bZIP positive cells (pink) among K-Rta expressing cells (red) was calculated as % of K-Rta positive cells. *; P<0.05 (TIFF) [file ppat.1005051.s010.tiff]

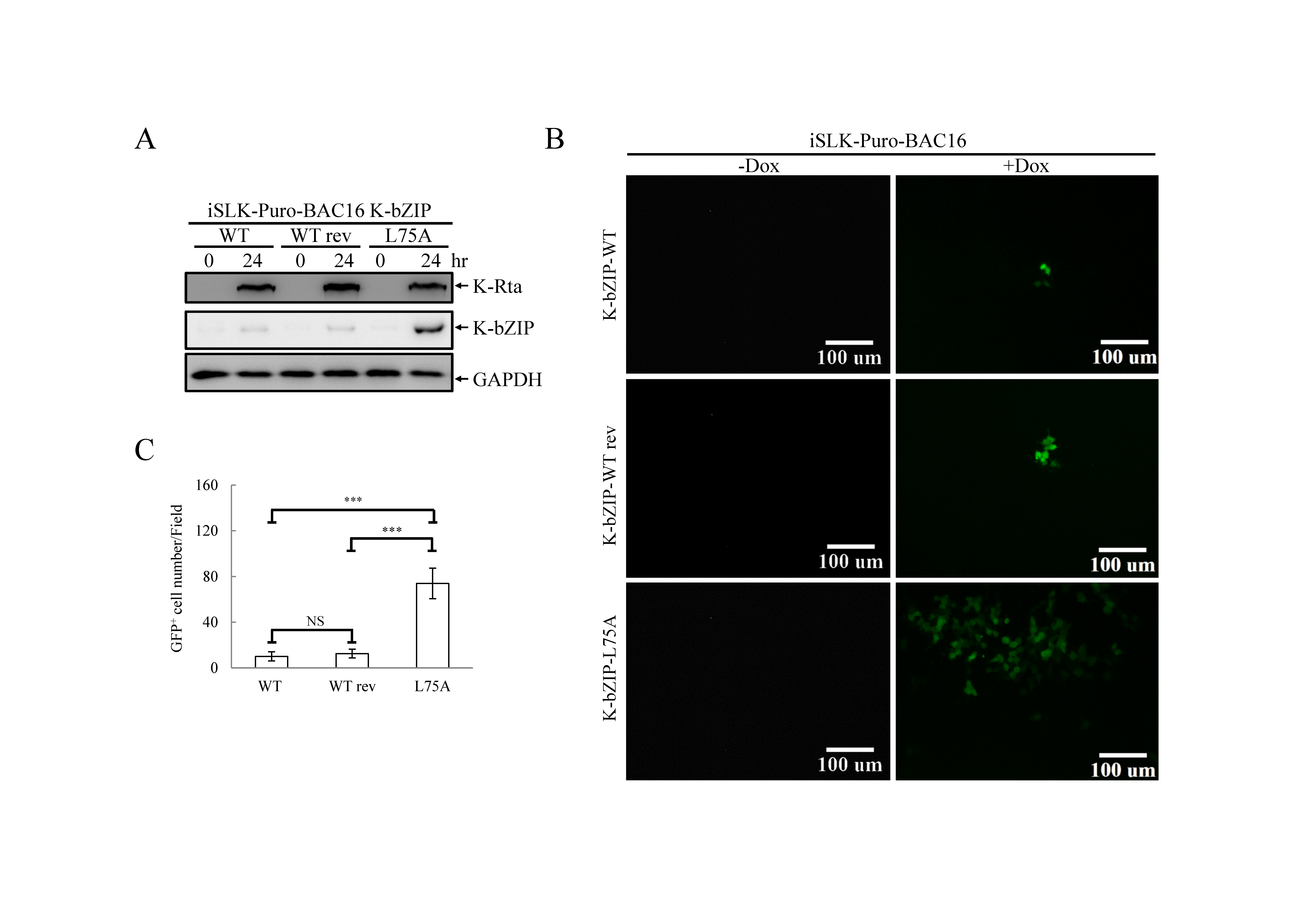

Supplement: S11 Fig — (A) The expression of K-Rta and K-bZIP in iSLK-Puro harboring parental BAC16 clone, iSLK-Puro-BAC16 K-bZIP-WT, and iSLK-Puro-BAC16 K-bZIP-WT rev and -L75A cells before and after Dox induction for 24 hours was analyzed by immunoblotting. GAPDH was probed as control. (B) Supernatants harvested from iSLK-Puro-BAC16 K-bZIP-WT, -WT rev and -L75A cells treated with or without Dox for 72 hours were filtered and used to infect 293T cells. GFP positive cells were analyzed by fluorescence microscopy (FITC, 10X magnification) 48 hours after infection. (C) The GFP positive cells were quantified using the average from >20 microscopic fields. ***; P<0.001. NS; non-significant. (TIFF) [file ppat.1005051.s011.tiff]

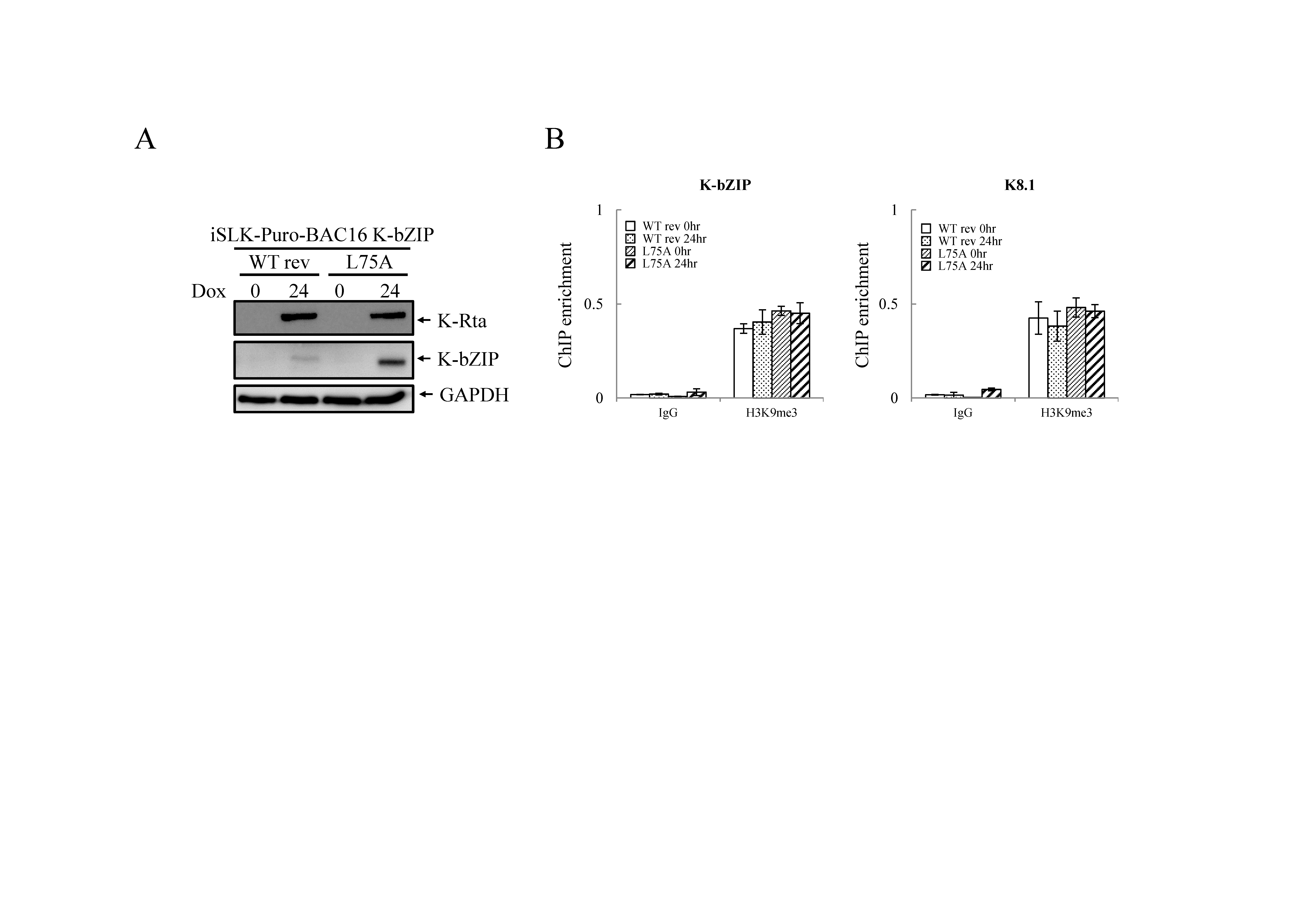

Supplement: S12 Fig — (A) TCLs were collected from iSLK-Puro-BAC16 K-bZIP-WT rev and -L75A cells treated as described in Fig 9 and analyzed using anti-K-Rta and anti-K-bZIP antibodies. Anti-GAPDH antibody was used for loading control. (B) ChIP was performed using chromatin prepared as described in (A) using anti-H3K9me3 antibody. Rabbit IgG was used as negative antibody control. (TIFF) [file ppat.1005051.s012.tiff]
