# Supplementary material for: K-bZIP Mediated SUMO-2/3 Specific Modification on the KSHV Genome Negatively Regulates Lytic Gene Expression and Viral Reactivation
Source: PLoS Pathog. 2015 Jul 21;11(7):e1005051. doi: 10.1371/journal.ppat.1005051 (PMC4510548; doi:10.1371/journal.ppat.1005051)
Supplement: S1 Table — (DOCX) [file ppat.1005051.s013.docx]

**Table S1.** Primer sequence for ChIP-qPCR

| Prime | Forward sequence | Reverse sequence |
| --- | --- | --- |
| orf19 | CCCGATACCAGAATTACGCA | GCCGAGGTTAAGAAATCCC |
| orf20 | GGCTACTTAGAAACCGCCA | TCGTCCATATCCGTGCTG |
| orf23 | AACATAGCTTAGACCAGCGG | AAGAAGACCAGGCTAGACGA |
| orf25 | TGTAGCTGTGCATCTGGG | GGATCATGGGAACTTACCGA |
| orf46 | GGAAATCCACTTAGGTGCCA | CAACGAAGTTTGACGGCCT |
| orf52 | GCGTTAATCAAGGCCCAGAC | TGGCCCTGGCTTTATTTGTG |
| K-bZIP | GATCAGTCACATTCTCCCAC | ACATTCGCATCAGCATGTC |
| K8.1 | GAGACTGAAGTGTTCGCAAGG | CGACAACGGAGGAAATACCA |
| RTP4 | GAAACTGAACCGAACACCGA | TGAAATGTCTGCTCCCAAGTC |
