# Supplementary material for: K-bZIP Mediated SUMO-2/3 Specific Modification on the KSHV Genome Negatively Regulates Lytic Gene Expression and Viral Reactivation
Source: PLoS Pathog. 2015 Jul 21;11(7):e1005051. doi: 10.1371/journal.ppat.1005051 (PMC4510548; doi:10.1371/journal.ppat.1005051)
Supplement: S2 Table — (DOCX) [file ppat.1005051.s014.docx]

**Table S2.** Primer sequence for RT-qPCR

| Prime | Forward sequence | Reverse sequence |
| --- | --- | --- |
| orf19 | ATACCAGGTTCAAGCGGCG | TGGATTGCTGGAGTTTGGG |
| orf20 | TACCAGAATTACGCAGTCGG | CGGCTCCTCAAGTACTCCA |
| orf23 | TGCCGTCACATATCAGTTCGA | CCCCAAAGACCGTCAAAGC |
| orf25 | CTCGGCGACGTGCTATACAAT | TGCCGACAAGGACTGTACATG |
| orf46 | TGAACCAATCCCAGCCAAG | GTTTTGACGGTGGAGAAGGG |
| orf52 | GGCACCAGGAGGCGGT | TCGCTTAGAATCGACGTCTGC |
| K-bZIP | GGTCTGTGAAACGGTCATTGA | TCTATGTAGTCGCCTCTTGGA |
| K8.1 | AAAGCGTCCAGGCCACCACAGA | GGCAGAAAATGGCACACGGTTAC |
| SUMO-2 | AAGATTAAGAGGCATACACCAC | TCATCCTCCATTTCCAACTG |
| SUMO-3 | AGAATGACCACATCAACCTG | GAGTGTCAGTTTCATTGATTGG |
